# Rearrangement Cascade Initiated by Nucleophilic Benzyne Attack on 3,6-Di(2-pyridyl)-1,2-diazines

**DOI:** 10.1021/acsorginorgau.4c00070

**Published:** 2025-01-28

**Authors:** Johannes Schöntag, Theresa Hettiger, William Roberts, Marcus Scheele, Markus Ströbele, Holger F. Bettinger

**Affiliations:** † Institut für Organische Chemie, Universität Tübingen, Auf der Morgenstelle 18, 72076 Tübingen, Germany; ‡ Institut für Physikalische und Theoretische Chemie, Universität Tübingen, Auf der Morgenstelle 18, 72076 Tübingen, Germany; § Institut für Anorganische Chemie, Universität Tübingen, Auf der Morgenstelle 18, 72076 Tübingen, Germany

**Keywords:** rearrangement, pyridotriazole, mechanistic study, aryne, nitrogen containing PAH

## Abstract

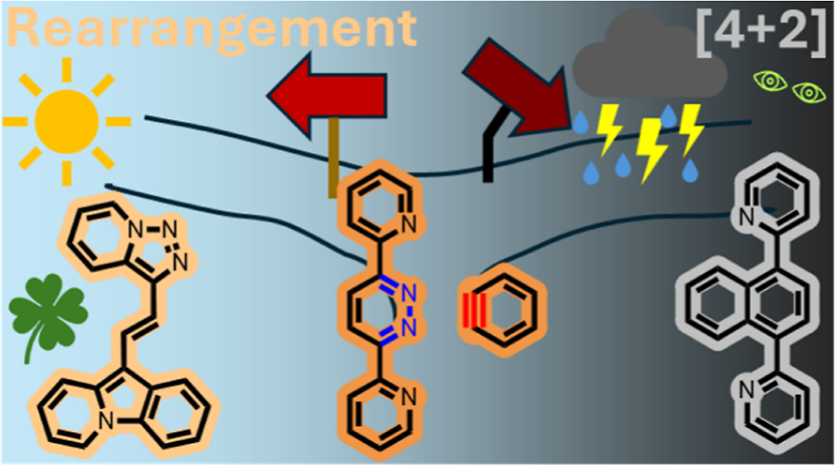

Aryne intermediates in synthetic organic chemistry offer versatile routes to complex heterocyclic structures that are valuable in pharmaceuticals and materials science. We present a one-step aryne-mediated reaction to synthesize pyrido[1,2-*a*]indoles interconnected through vinylene or 1,2-phenylene linkers to pyridotriazoles using 2-pyridyl-substituted pyridazines and phthalazines as confirmed via single-crystal X-ray crystallography and NMR spectroscopy. This unexpected rearrangement proceeds under mild conditions. Considering that five bonds are broken and three new bonds are formed in the reaction between 3,6-di-2-pyridyl-1,2,4,5-tetrazine and benzyne, the yield of 16% is fair. Electron-rich substituents on aryne precursors destabilized the products, while electron-deficient substituents offered some stability improvements. DFT studies could reveal the mechanism of this rearrangement.

## Introduction

The reactivity of aryne intermediates in modern synthetic organic chemistry offers versatile routes to complex heterocyclic structures.^1−3^ Aryne reactions have found wide-ranging applications across various fields, from pharmaceuticals^2,4^ to materials science,^5^ owing to their efficient engagement in cycloadditions and other reactions. Recently, significant attention has been directed toward pyrido[1,2-*a*]indoles^6^ and pyridotriazoles.^7,8^ Pyrido[1,2-*a*]indoles are prevalent in natural products and pharmaceuticals, exhibiting a spectrum of bioactivities including cytotoxicity and receptor affinity,^9−11^ along with interesting properties for materials science.^12,13^ Pyridotriazoles, encompassing a broad class of nitrogen-containing heterocycles, are pivotal in medicinal chemistry and materials science due to their pharmacological relevance and functional versatility.^7,14^

**Scheme 1:**
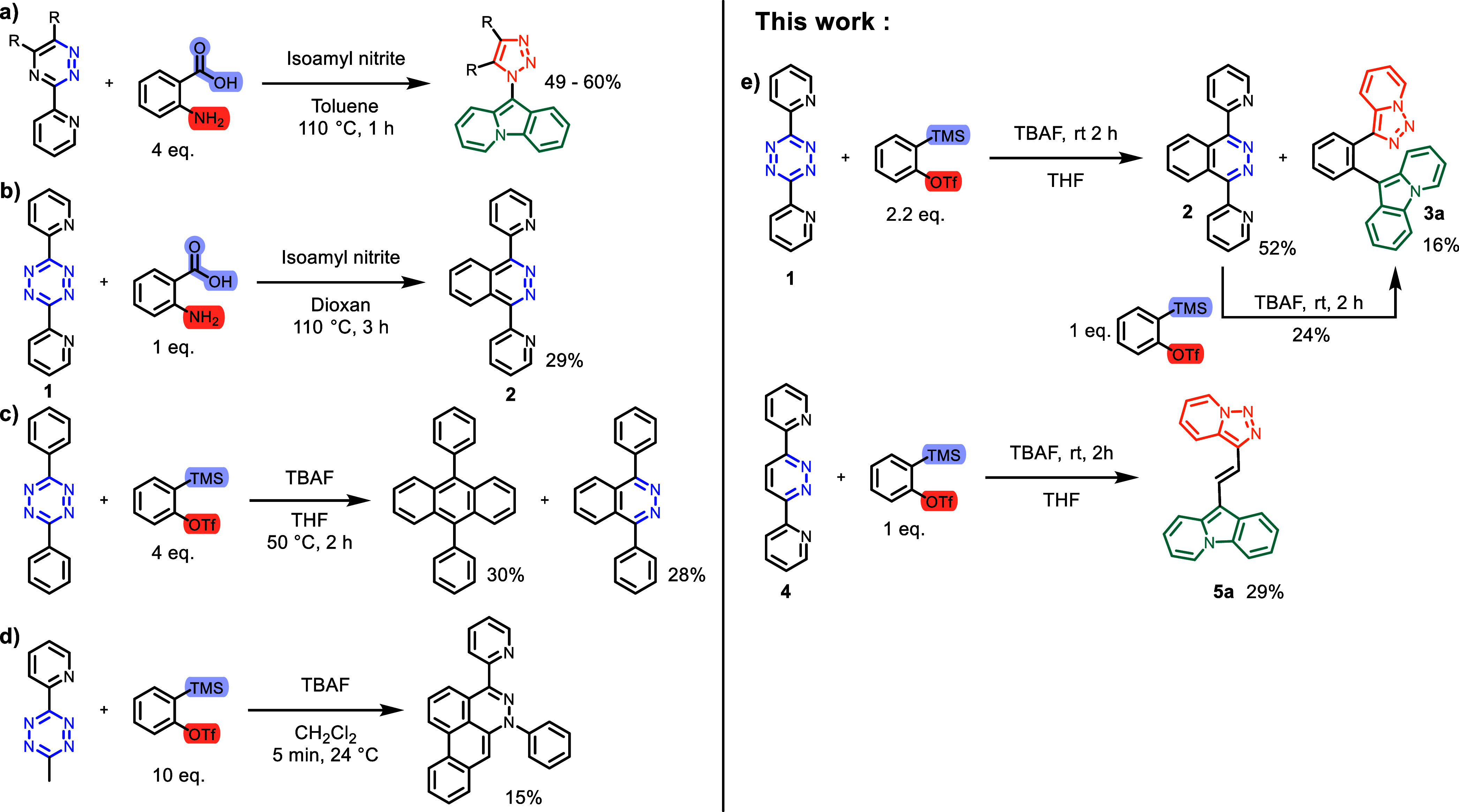
(a) Rearrangement Reaction of Pyridine-Substituted Triazine to Pyridotriazole Reported by Chupakhin et al.^15^ (b) Synthesis of Pyridine-Substituted Phthalazine Reported by Margetić et al.^18^ (c) Synthesis of Phenyl-Substituted Anthracene and Phthalazine Derivatives Reported by Chenoweth et al.^19^ (d) Triple Aryne Reaction from 3-Methyl-6-(2-pyridyl)-1,2,4,5-tetrazine to Dibenzo[*de*,*g*]cinnolines.^19,20^ (e) Our Work, Rearrangement Reaction via Ring Opening of the Pyridazine or Phthalazine Ring.

The formation of molecules containing the 10-(1*H*-1,2,3-triazol-1-yl)pyrido[1,2-*a*]indole motif was observed to result from reactions of 3-(2-pyridyl)-1,2,4-triazines with arynes (Scheme 1a).^15−17^ The products hint toward the interaction of the aryne with the pyridine nitrogen atom.^15^ On the other hand, 3,6-di(2-pyridyl)-1,2,4,5-tetrazine **1** is known to react with one equivalent of benzyne to 1,4-di(2-pyridyl)phthalazine **2** by inverse electron demand Diels–Alder (IEDDA) cycloaddition and N_2_ cycloreversion (Scheme 1b), and no additional products were described by the authors.^18^ In the case of 3,6-diphenyl-1,2,4,5-tetrazine, this reaction yields a mixture of 1,4-diphenylphthalazine and 9,10-diphenylanthracene, the latter by a second sequence of Diels–Alder–*retro*-Diels–Alder reactions (Scheme 1c).^19^ 3-Methyl-6-(2-pyridyl)-1,2,4,5-tetrazine undergoes a complex reaction to a polycyclic heteroaromatic system, but it does not appear that the 2-pyridyl group is involved in product formation (Scheme 1d).^19,20^

In view of these literature reports, we investigated the reaction between **1** and an excess of benzyne to elucidate its tendency to undergo a second IEDDA reaction or an interaction with the nucleophilic 2-pyridyl groups. We found a new one-step aryne reaction with 2-pyridyl-substituted tetrazines, phthalazines, and pyridazines that gives pyrido[1,2-*a*]indoles (green) linked to pyridotriazoles (orange) by a phenylene or vinylene spacer within a single molecular framework (Scheme 1e). This transformation occurs under mild conditions, providing easy access to these complex heterocyclic compounds from commercially available starting materials.

## Results and Discussion

Treatment of **1** with 2.2 equiv of benzyne that was generated in situ from 2-(trimethylsilyl)phenyl triflate and TBAF in THF gave a mixture of known **2** and unknown **3a** (Scheme 1e). The two compounds could be separated by column chromatography. Treatment of **2** with 1 equiv of aryne precursor and TBAF gave **3a**. This shows that the new product **3a** is a follow-up product of the first formed Diels–Alder–*retro*-Diels–Alder product **2**. We have not found evidence for the formation of 9,10-di(2-pyridyl)anthracene that could be expected to result from **2** by a second Diels–Alder–*retro*-Diels–Alder sequence.

**Figure 1:**
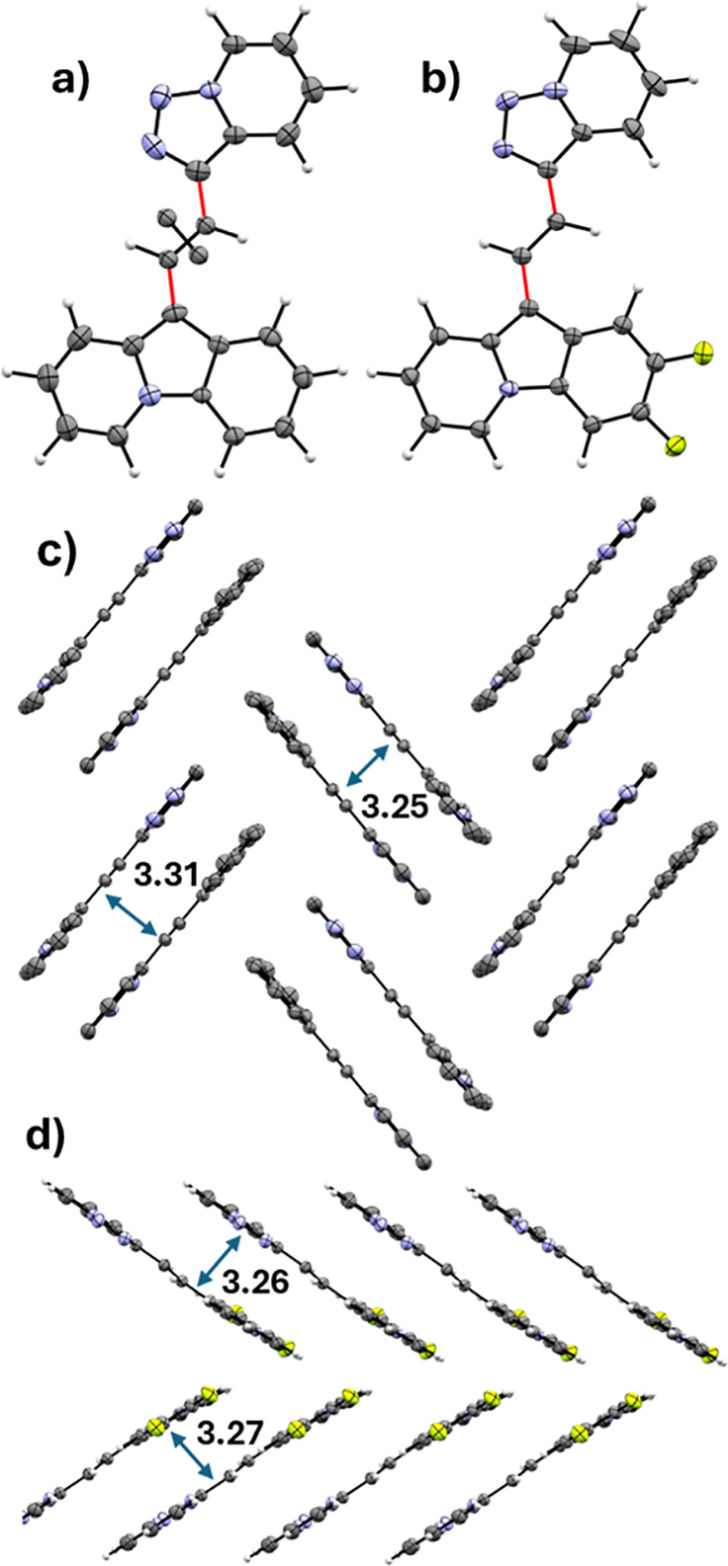
(a) Molecular structure of **5** in the solid state, (b) molecular structure of **5c** in the solid state, (c) sandwich herringbone packing of **5**, and (d) γ-packing of **5c**.^23^ Anisotropic displacement parameters are depicted at the 50% probability level.

As phthalazine **2** reacted with benzyne to give **3a**, we also investigated the reaction of pyridazine analogue **4** and found that it gave unknown compound **5a** (Scheme 1e). The identity of **5a** could be proven by single-crystal X-ray crystallography (Figure 1), NMR, and high-resolution mass spectrometry (HRMS). The NMR spectral data of **3a** is similar to that of **5a**, allowing the assignment to the structure displayed in Scheme 1e.

A change of the temperature or fluoride source (CsF, KF/18-crown-6 ether) did not increase the yields of **3a** and **5a**. Even increasing the equivalents of the aryne precursor did not seem to have an impact on yields. It was always possible to recover significant amounts of the unreacted starting material [pyridazine **4** (60%) and tetrazine **1** (29%)], while it was not possible to recover the aryne precursor. Possibly the products react more readily with benzyne than with the educt. Products **3a** and **5a** must be stored under inert gas conditions as otherwise they decompose within a few days.

**Scheme 2:**
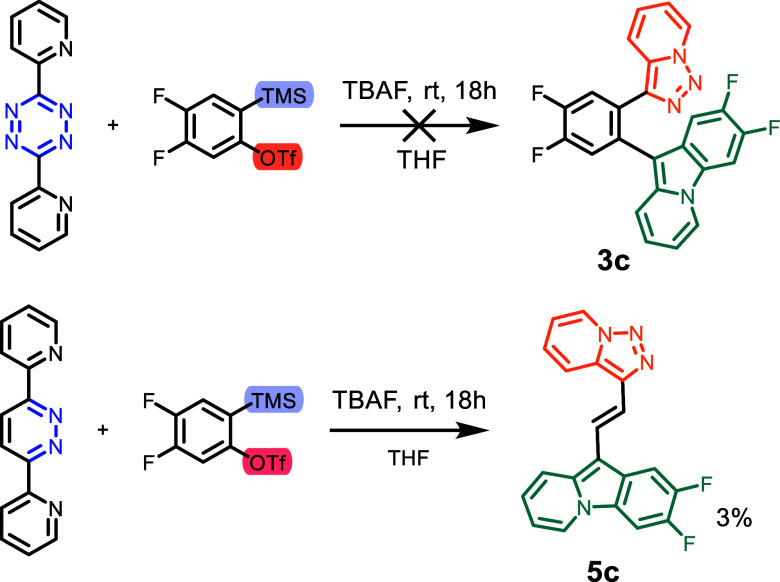
Synthesis Attempts of Electron-Poor Derivatives of **3** and **5** with Doubly Fluorinated Aryne Precursor (**3c** and **5c**)

We attempted to synthesize derivatives of **3a** and **5a** with the goal of tuning their properties, utilizing various benzyne precursors in the process. Mono- and dimethoxy-substituted aryne precursors were reacted with pyridazine **4**. A mono-methoxy-substituted pyridotriazole **5b** could be detected via HRMS, but it decomposed quickly in the purification process and could only be obtained in an impure form. Even in the glovebox in the dark, it decomposed in solution, and thus crystallization attempts failed. Addition of 4,5-dimethoxy benzyne to 3-(pyridin-2-yl)-1,2,4-triazines also led to lower yields in the rearrangement reaction reported by Chupakhin et al.^21^ Apparently, adding electron-donating groups onto **5a** does not benefit its stability; thus, we did not attempt to introduce these substituents to **3a**. Attempts to add electron-deficient benzynes to **4** and **1** did lead to significantly lower or no yields (Scheme 2). No evidence of the formation of **3c** could be found. **5c** could be isolated in only 3% yield. Similarly, Chupakhin et al. reported that the addition of 4,5-difluoro benzyne to 3-(pyridin-2-yl)-1,2,4-triazines resulted in lower yields in the rearrangement reaction.^22^ Still, it appears that **5c** is more stable than the nonfluorinated pyridotriazole **5a**.

The identities of novel compounds **3a**, **5a**, and **5c** were verified by NMR and HRMS. For compounds **5a** and **5c**, single crystals could be grown by evaporation of a solution (DCM/*n*-hexane) of the corresponding compound inside a small vial under inert gas conditions. The X-ray analysis shows an almost planar structure for **5a** (Figure 1a,c). The highlighted single bonds in red are shorter than usual (1.44 and 1.46 Å), which hints toward a conjugated system. The crystal contains a crystallographic defect, as in 1/3 of the molecules the central double bonds are mirrored. The molecules pack in a sandwich herringbone^23^ motif with distances between the molecules of 3.31 and 3.25 Å. Compound **5c** is also planar in analogy to its parent compound **5a**. Similarly, the highlighted bonds in red are shorter (1.44 and 1.45 Å) than usual, which again speaks for the conjugation of the system. The packing of the molecules in the solid state changes from sandwich herringbone to γ-configuration^23^ upon introduction of two fluorine substituents. The distances between the layers are 3.26 and 3.27 Å, respectively. Although it was not possible to grow a single crystal suitable for X-ray analysis of compound **3a**, the similarity of the NMR spectra of **3a** and **5a** allows structural assignment.

**Figure 2:**
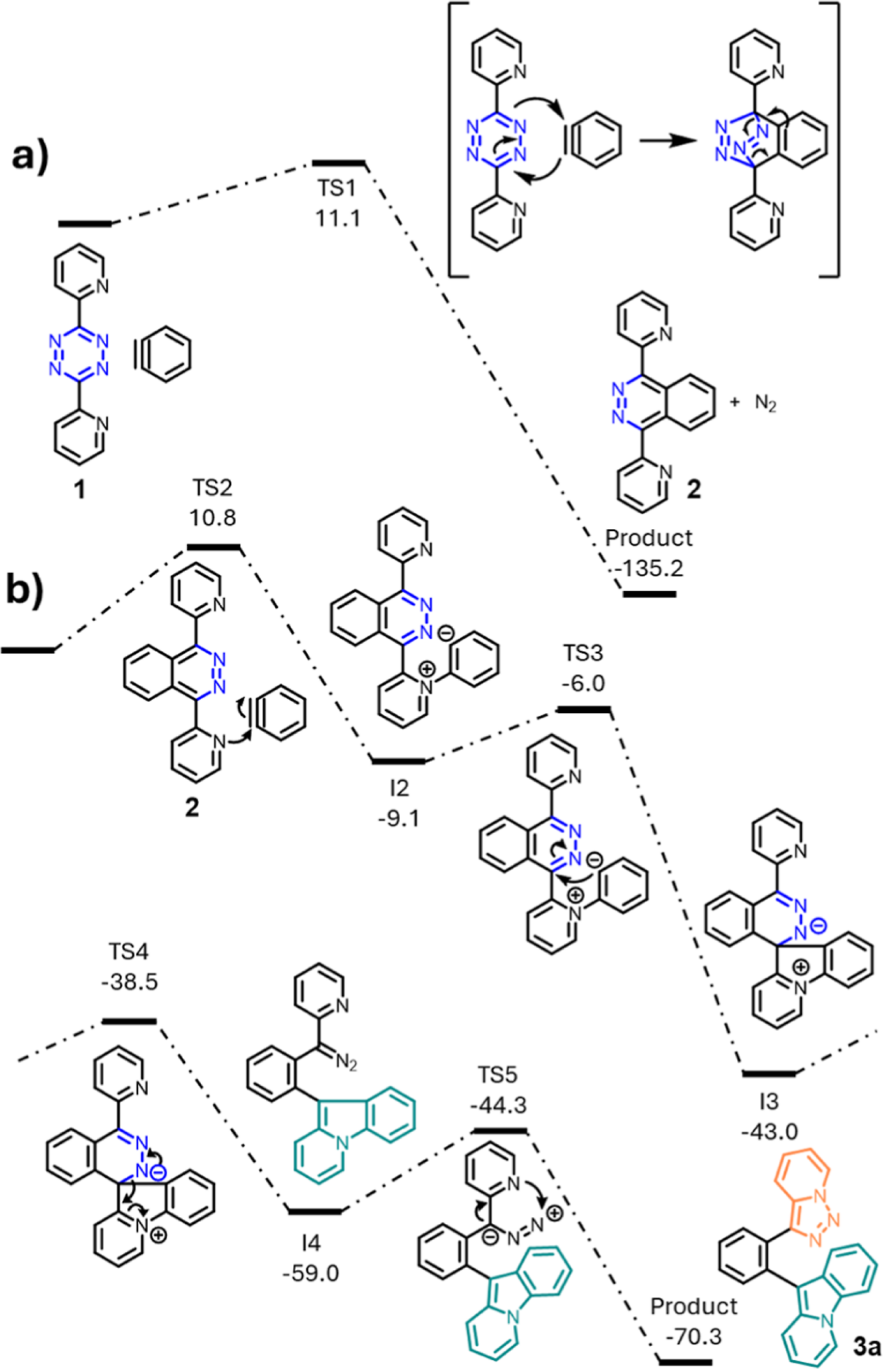
(a) Relative Gibbs energies (calculated at 298 K, kcal/mol) computed (M06-2X/6-311+G**/SMD(THF)) for the initial Diels–Alder–*retro*-Diels–Alder reaction to yield phthalazine **2** and (b) rearrangement of phthalazine **2** to pyridotriazole **3a**.

**Figure 3:**
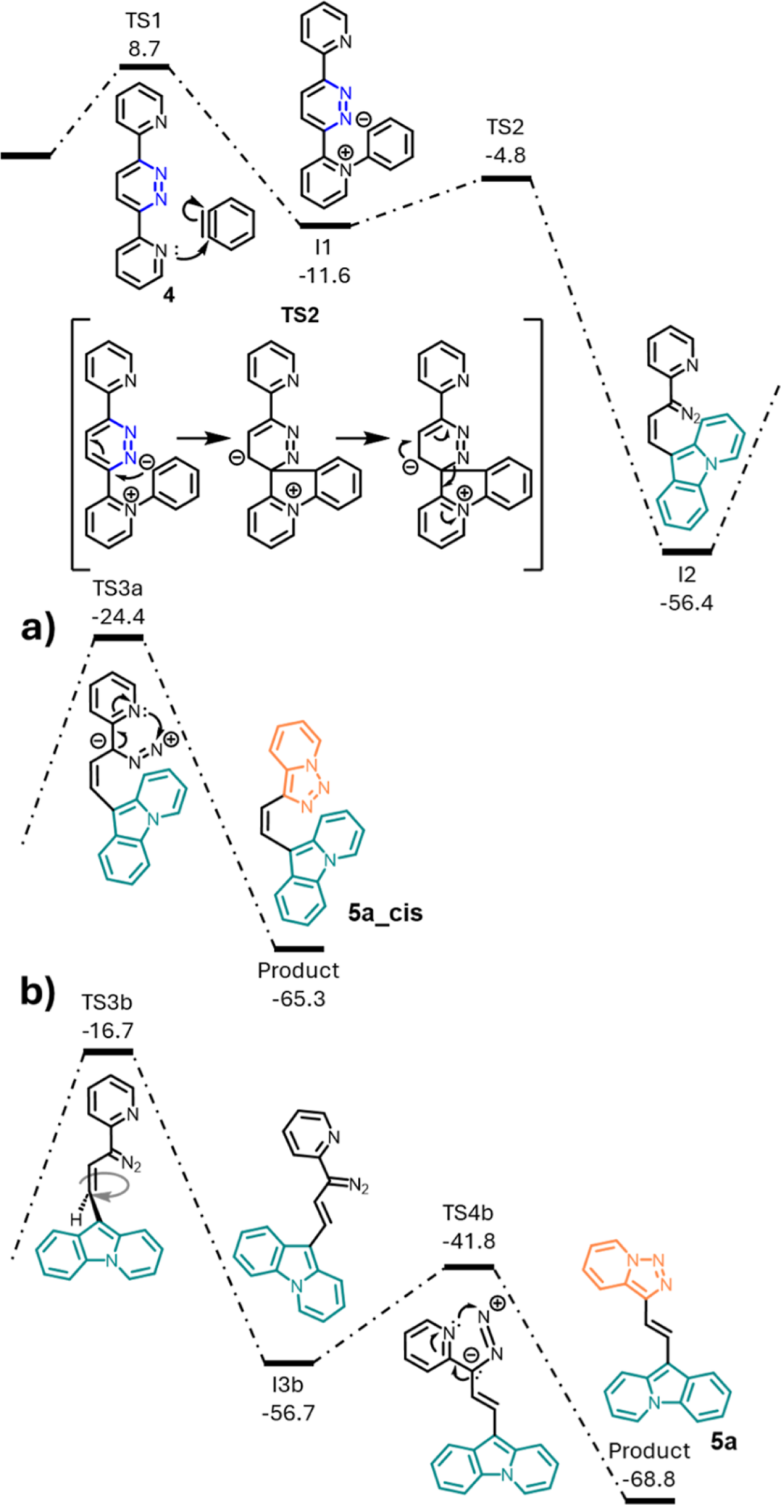
Energy profiles calculated (M06-2X/6-311+G**/SMD(THF)) for the rearrangement of pyridazine **4** to pyridotriazole **5a**: (a) *cis* configuration and (b) *trans* configuration. Relative Gibbs energies (calculated at 298 K) are given in kcal/mol. TS3b was calculated with UM06-2*X*/6-311+G**/SMD(THF).

The reaction mechanism of the rearrangement leading to **3a** and **5a** was investigated by DFT computations (Figures 2 and 3). Figure 2a shows the initial Diels–Alder–*retro*-Diels–Alder reaction to yield phthalazine **2**. This stable intermediate engages in a nucleophilic attack on the benzyne triple bond (Figure 2b, TS2), leading to a zwitterionic intermediate (I2). Ring closure (TS3) of I2 leads to another zwitterionic intermediate (I3) followed by subsequent C–N bond breaking (TS4) to yield diazo intermediate I4. This diazo intermediate I4 undergoes a chain-ring isomerization (TS5) to form pyridotriazole **3a** by nucleophilic attack of the pyridine on the terminal nitrogen atom of the diazo unit. The barrier heights for the initial IEDDA reaction between **1** and benzyne (TS1, 11.1 kcal/mol) and the nucleophilic addition of **2** to benzyne (TS2, 10.8 kcal/mol) are similar. The highest barrier toward the formation of product **3a** is the attack of the second pyridyl group on the terminal nitrogen atom of the diazo group (TS5, 14.7 kcal/mol).

In the case of pyridazine **4** (Figure 3), the barrier of the nucleophilic attack (TS 1) is slightly lower (by 2.1 kcal/mol), and the resulting intermediate I1 is slightly more stable (by 2.5 kcal/mol) than computed for the reaction of phthalazine **2**. In the following transition state (TS2), the ring closure leads to an immediate C–N bond breaking in a single step, which gives diazo intermediate I2 in the *cis* configuration. From this *cis* intermediate, a ring closure is possible, but it has a very high barrier (TS3a, 32.0 kcal/mol). The resulting product would be **5a_cis** (Figure 3a). In the X-ray analysis of **5a**, only the *trans* isomer is present and its solution ^1^H NMR also hints solely toward the *trans* isomer, as indicated by the coupling constant between vinylic protons (16 Hz). The *cis*/*trans* isomerization of the final product is unlikely as the computed corresponding transition state is very high in energy (45.1 kcal/mol, calculated with UM06-2*X*/6-311+G**, as M06-2X could not find a transition state). The *cis*/*trans* isomerization of intermediate I2 also has a sizable barrier (TS3b, 39.7 kcal/mol, UM06-2*X*/6-311+G**). The same TS calculated with the UB3LYP functional yields a barrier of 49.7 kcal/mol. This process would result in *trans* intermediate I3b, and the resulting ring closure reaction to yield the final product has a barrier of only 15.1 kcal/mol (TS4b). The origin of this isomerization is unclear. Possibly a catalytic process may provide a lower barrier pathway.

**Scheme 3:**
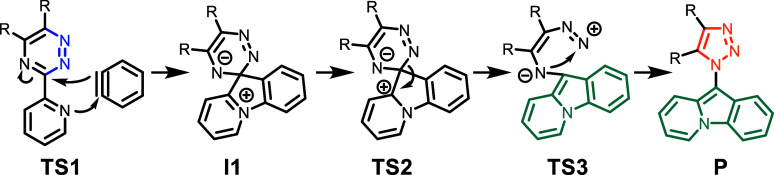
Mechanism for the Rearrangement of 3-(2-Pyridyl)-1,2,4-triazines with Benzyne to 10-(1*H*-1,2,3-Triazol-1-yl)pyrido[1,2-*a*]indole Proposed by Chupakhin et al.

The mechanisms we computed are similar to that suggested by Chupakhin et al.^15^ (Scheme 3) for the rearrangement observed in the reaction of 3-(2-pyridyl)-1,2,4-triazines and benzyne (Scheme 1a). The nucleophilic attack of the first pyridine substituent on the triple bond of benzyne (TS1) and the ring closure to pyrido[1,2-*a*]indole (TS2) are comparable. The second ring closure reaction (TS3) is different as the third triazine nitrogen is missing in our case and the nitrogen of the second pyridine substituent is attacked instead.

Additional paths that lead to different products were also discovered (Figure S3). The mechanisms in Figure S3a–c would form a nitrogen-substituted triphenylene subunit. The barriers from the first intermediates I1/I3/I5 to these products are significantly higher than those that lead to the formation of **3a** and **5a**. We could not gain any evidence for the formation of these *N*-triphenylene products. Moreover, Diels–Alder reactions with the pyridazine rings are conceivable (Figure S3d,e). These reactions also have higher barriers, and neither the naphthalene nor the anthracene derivatives were detected. The nucleophilic attack of either nitrogen (Figure S3a–c) is favored by roughly 7–8 kcal/mol over the Diels–Alder reaction (Figures S3d and S4e).

**Figure 4:**
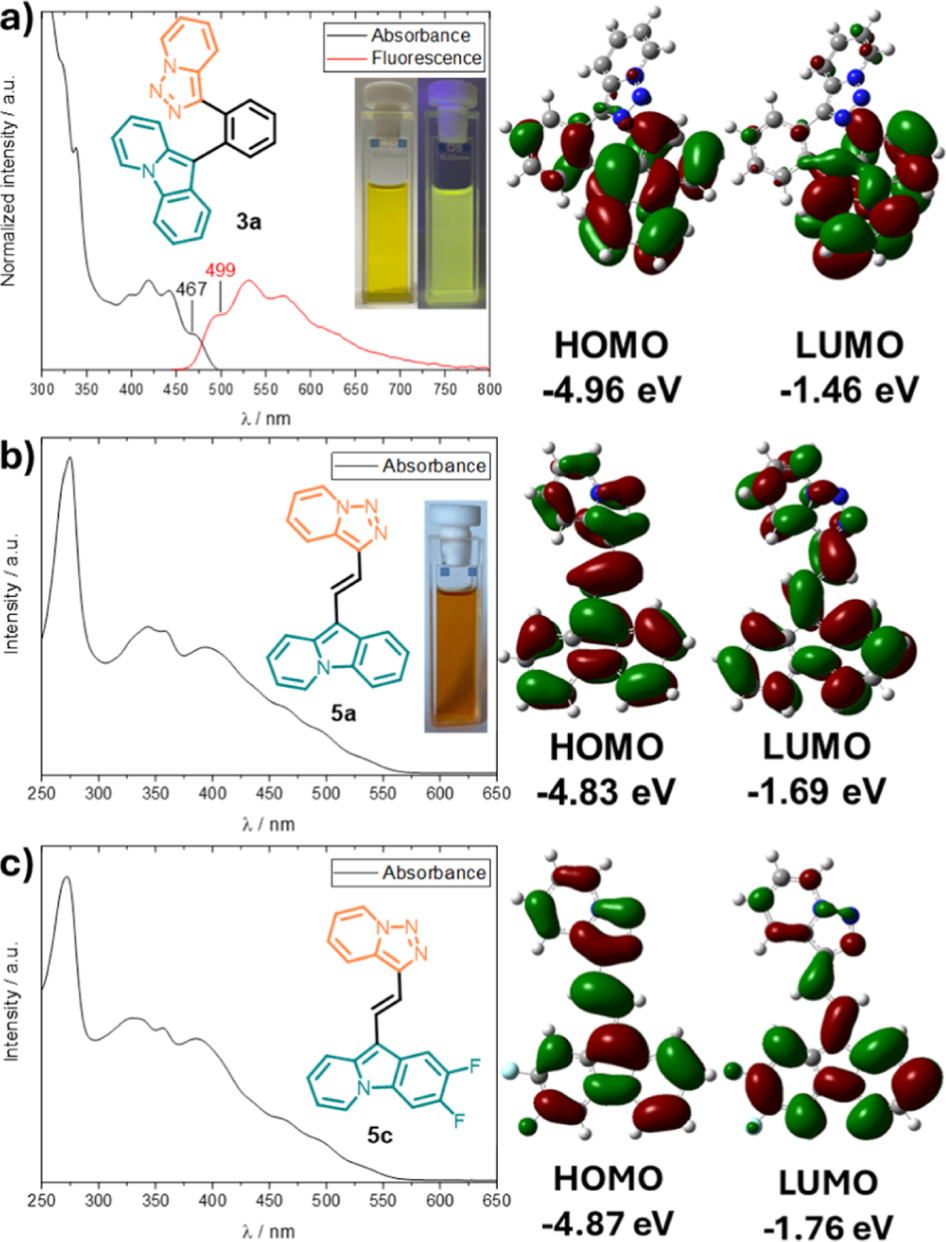
Optical spectra (1 × 10^–4^ M, DCM) and calculated HOMO–LUMO Frontier orbitals (M06-2X/6-311+G**/SMD(THF)) with their respective energies (B3LYP/6-311+G**, SMD = THF): (a) **3a** (λ(exc.) = 300 nm), (b) **5a**, and (c) **5c**.

The electronic absorption spectrum of **3a** shows three maxima in the visible range at 442, 419, and 399 nm, a shoulder at 467 nm, and an absorption onset at around 500 nm (Figure 4a). The fluorescence spectrum of **3a** is the mirror image of the absorption spectrum with a Stokes shift of 1373 cm^–1^ (0.17 eV), determined between the shoulders in the respective spectra. The absorption spectra of **5a** and **5c** are similar but differ from that of **3a** by further extension into the visible spectrum with absorption onsets at roughly 575 nm. In addition, the spectra are broader with several maxima (**5a**: 394, 358, 343, and 328 nm; **5c**: 386, 357, and 332 nm) and shoulders (**5a**: 529, 489, 458, and 434 nm; **5c**: 528, 489, 457, 433 nm). Also, neither **5a** nor **5c** shows fluorescence. A reason for the difference compared to **3a** could be the increased rotational flexibility involving the C–C single bonds between the heterocycles and the vinylene linker. The HOMO and LUMO of **3a**, **5a**, and **5c** reveal no strong charge separation in the excited state of all molecules (Figure 4a–c).

**Figure 5:**
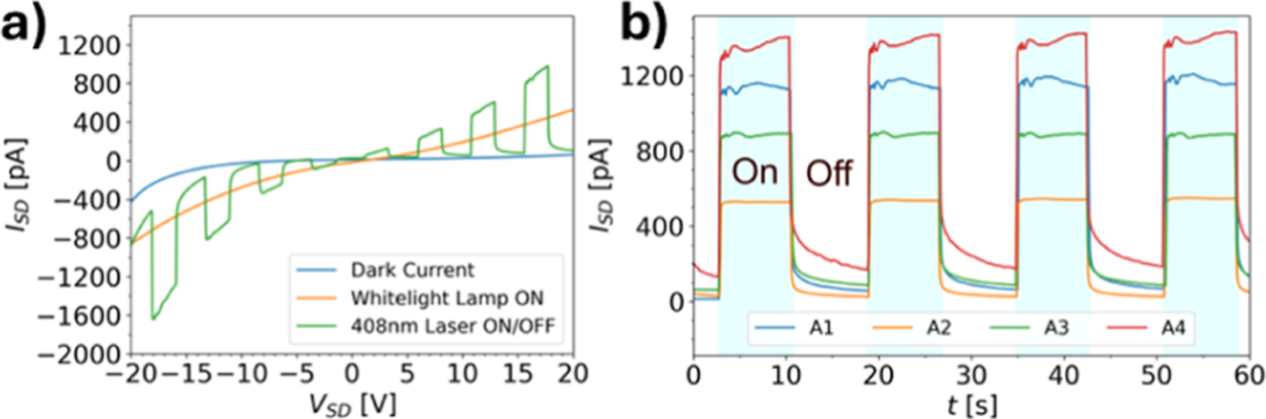
(a) Typical *I*–*V*-curves of a thin film of **5a** under various lighting conditions. (b) Photocurrent measurements of four **5a** thin film devices (A1–4) under illumination with a λ = 408 nm laser diode.

The half-wave potential of the oxidation was determined by cyclic voltammetry as +0.23 V for **3a** and −0.14 V for **5a** vs Fc/Fc^+^ (Figure S1). These low oxidation potentials show that **3a** and **5a** are electron-rich polycyclic aromatic hydrocarbons, consistent with the observed decomposition under ambient conditions and the faster degradation of **5a**. To gauge the suitability of **5a** for optoelectronic applications, we fabricate thin films via spin-coating onto silicon dioxide substrates with prepatterned gold electrodes. The films exhibit a weak conductivity in the dark (σ ≈ 10^–9^ to 10^–7^ S/m), which increases significantly under daylight (Figure 5a). Excitation with a 408 nm laser diode (*P*_opt_ ≈ 150 μW, *E*_e_ ≈ 0.3 W/cm^2^) results in a prompt photocurrent response with a typical responsivity of *R* ≈ 1.4 × 10^–5^ A/W and an ON/OFF ratio of 18 (Figure 5b). The fall times are slower, presumably due to the trapping of photoexcited carriers, in line with the negligible fluorescence from this compound.

## Conclusions

We discovered a one-step reaction via aryne chemistry to synthesize pyrido[1,2-*a*]indoles interconnected by 1,2-phenylene or vinylene linkers to pyridotriazoles from pyridine-substituted pyridazines and phthalazines. This unexpected rearrangement was studied by DFT computations, which clarified the nucleophilic attack and subsequent rearrangement steps. Electron-rich substitution tended to destabilize the products, while electron-deficient ones offered limited improvements as the stability increased but the yield decreased. Our findings introduce a new reaction pathway in aryne chemistry, providing a straightforward route to complex heterocyclic structures. We studied the half-wave potential of the oxidation of **3a** and **5a** and the photocurrent of **5a** in thin film devices. Further research could aim at increasing the yield, possibly by applying a flow chemistry system and evaluating the properties and potential applications of these compounds in pharmaceuticals and materials science.

## Experimental Section

### Triazolopyridine **3a**

3,6-Di-2-pyridyl-1,2,4,5-tetrazine (50 mg, 0.212 mmol) and 2-(trimethylsilyl)phenyl triflate (113 μL, 0.466 mmol) were dissolved in 10 mL of dry THF. Under stirring, TBAF (1 mol/L, 465 μL, 0.465 mmol) was slowly (5 min) added. The mixture immediately turned to a dark brownish color. After 2 h of stirring, water (1 mL) was added and extracted with DCM. The organic layer was dried with MgSO_4_, the solvent was evaporated, and the crude product was purified by column chromatography (SiO_2_, DCM/EtOAc 19:1, *R*_f_ = 0.67 DCM/EtOAc 9:1) to obtain a yellow solid (12.2 mg, 16%), (29% tetrazine and 52% phthalazine regained).

HRMS (ESI) *m*/*z*: [M + H]^+^ calculated for C_24_H_17_N_4_, 361.1448; found, 361.1449.

^1^H NMR (400 MHz, CDCl_3_): δ 8.43–8.41 (m, 1H), 8.17–8.15 (m, 1H), 8.01–7.98 (m, 1H), 7.77–7.72 (m, 2H), 7.67–7.65 (m, 1H), 7.58–7.50 (m, 2H), 7.29–7.20 (m, 2H), 7.11–7.08 (m, 1H), 6.68–6.64 (m, 1H), 6.58–6.54 (m, 1H), 6.46–6.40 (m, 2H), 6.35–6.31 (m, 1H).

^13^C NMR{^1^H} (100 MHz, CDCl_3_): δ 139.2, 133.4, 133.3, 132.0, 132.0, 131.0, 130.8, 129.3, 128.7, 127.9, 127.1, 124.8, 124.2, 123.8, 123.4, 122.9, 120.1, 119.1, 118.0, 117.8, 114.7, 110.1, 108.1, 104.7.

UV/vis (DCM, rt, 1 × 10^–4^ M, nm): λ_max_ = 467, 442, 419, 399, 375, 338, 321, 284, 266.

mp 90 °C.

### Triazolopyridine **5a**

3,6-Di(2-pyridyl)pyridazine (50 mg, 0.213 mmol) and 2-(trimethylsilyl)phenyl triflate (51.8 μL, 0.213 mmol) were dissolved in 3 mL of dry THF. Under stirring, TBAF (1 mol/L, 256 μL, 0.256 mmol) was slowly (5 min) added. The mixture immediately turned to a dark brownish color. After 2 h of stirring, water (1 mL) was added and extracted with DCM. The organic layer was dried with MgSO_4_, the solvent was evaporated, and the crude product was purified by column chromatography (SiO_2_, DCM/EtOAc 9:1, *R*_f_ = 0.69 DCM/EtOAc 4:1) to obtain a red solid (19 mg, 29%), (60% pyridazine regained).

HRMS (ESI) *m*/*z*: [M + H]^+^ calculated for C_20_H_15_N_4_ 311.1291; found 311.1291.

^1^H NMR (400 MHz, CDCl_3_): δ 8.70–8.68 (m, 1H), 8.34–8.32 (m, 1H), 8.28–8.26 (m, 1H), 8.09–8.05 (d, *J* = 16.5 Hz, 1H), 7.95–7.89 (m, 2H), 7.82–7.80 (m, 1H), 7.54–7.50 (m, 1H), 7.40–7.36 (m, 2H), 7.25–7.22 (m, 1H), 7.06–7.02 (m, 1H), 6.98–6.94 (m, 1H), 6.60–6.56 (m, 1H).

^13^C NMR{^1^H} (100 MHz, CDCl_3_): δ 138.7, 135.9, 130.4, 130.0, 127.0, 125.7, 125.6, 124.7, 124.4, 124.1, 123.9, 122.2, 120.9, 120.6, 118.4, 188.2, 115.4, 110.8, 110.6, 109.2.

UV/vis (DCM, rt, 1 × 10^–4^ M, nm): λ_max_ = 529, 489, 458, 434, 394, 358, 343, 328, 275.

mp 170 °C (decomp).

### Triazolopyridine **5c**

Same procedure as that for **5a** using 3,6-di(2-pyrididyl)pyridazine (50 mg, 0.213 mol), 4,5-difluoro-2-(trimethylsilyl)phenyl trifluoromethanesulfonate (54.8 μL, 0.213 mol), and TBAF (1 mol/L, 256 μL, 0.256 mol) in dry THF (3 mL).

**5c**, red solid, (5 mg, 3%) (SiO_2_, DCM/EtOAc 29:1, *R*_f_ = 0.43 DCM/EtOAc 19:1).

HRMS (ESI) *m*/*z*: [M + H]^+^ calculated for C_20_H_13_F_2_N_4_, 347.1103; found, 347.1107.

^1^H NMR (400 MHz, CDCl_3_): δ 8.72–8.70 (m, 1H), 8.17–8.15 (m,1H), 8.02–7.92 (m, 3H), 7.81–7.78 (m, 1H), 7.70–7.66 (m, 1H), 7.30–7.23 (m, 2H), 7.06–7.02 (m, 1H), 7.00–6.97 (m, 1H), 6.63–6.60 (m, 1H).

^13^C NMR{^1^H} (176 MHz, CDCl_3_): δ 149.2 (dd, *J* = 244, 15 Hz), 147.0 (dd, *J* = 244, 16 Hz), 138.2, 136.8 (d, *J* = 3 Hz), 130.2, 125.6, 125.3 (d, *J* = 9 Hz), 124.7, 124.4, 123.7, 122.5 (d, *J* = 8 Hz), 121.4, 118.3, 118.2, 115.5, 111.1, 109.9, 107.1 (d, *J* = 20 Hz), 103.0 (d, *J* = 4 Hz), 98.9 (d, *J* = 22 Hz).

^19^F NMR (377 MHz, CDCl_3_): δ −140.39 (ddd, *J* = 6.8, 11.5, 20.8 Hz), −142.60 (ddd, *J* = 7.5, 10.2, 20.5 Hz).

UV/vis (DCM, rt, 1 × 10^–4^ M, nm): λ_max_ = 528, 489, 457, 433, 386, 357, 332, 272.

mp 174 °C (decomp).

**5b**, red solid, (8 mg, impure) (SiO_2_, DCM/EtOAc 9:1, *R*_f_ = 0.38 DCM/EtOAc 9:1).

HRMS (ESI) *m*/*z*: [M + H]^+^ calculated for C_21_H_16_N_4_O, 341.1497; measured 341.1402.

## Data Availability

The data underlying this study are available in the published article, in its Supporting Information, and openly available in RADAR4Chem at 10.22000/njxqt7bv0n7vdsvk.

## Supporting Information Available

Experimental details—general, optical measurements, cyclic voltammetry, thin film fabrication, photocurrent measurements, and computational methods; and experimental data—crystallographic data, ^1^H and ^13^C NMR spectra of all compounds, high-resolution mass spectra, computational data, and Cartesian coordinates (PDF)
