# Supplementary material for: Rearrangement Cascade Initiated by Nucleophilic Benzyne Attack on 3,6-Di(2-pyridyl)-1,2-diazines
Source: ACS Org Inorg Au. 2025 Jan 28;5(2):97–104. doi: 10.1021/acsorginorgau.4c00070 (PMC11969272; doi:10.1021/acsorginorgau.4c00070)
Supplement: Supplementary file 1 — gg4c00070_si_001.pdf [file gg4c00070_si_001.pdf]

Supporting Information

Rearrangement Cascade Initiated by Nucleophilic Benzyne Attack on 3,6-Di(2-pyridyl)-1,2-diazines

Johannes Schöntag[a], Theresa Hettiger[b], William Roberts[b], Marcus Scheele[b], Markus Ströbele[c], Holger F. Bettinger[a]*

[a] Institut für Organische Chemie, Universität Tübingen, Auf der Morgenstelle 18, 72076 Tübingen, Germany, E-mail: holger.bettinger@uni-tuebingen.de

[b] Institut für Physikalische und Theoretische Chemie, Universität Tübingen, Auf der Morgenstelle 18, 72076 Tübingen, Germany

[c] Institut für Anorganische Chemie, Universität Tübingen, Auf der Morgenstelle 18, 72076 Tübingen, Germany

Table of Contents

General 1

Optical measurements 2

Cyclic voltammetry (CV) 2

Thin Film Fabrication 3

Dark and Photocurrent measurements 4

Computational Methods 4

Crystallographic data 5

5a 5

5c 6

NMR 8

Mass spectrometry 12

Computational data 14

General

All reactions have been performed under inert gas conditions. THF has been dried over a SPS-800 from *Braun*. Chemicals were commercially bought from *Acros*, *Sigma-Aldrich*, *TCI Chemicals* and *fluorochem* and used without further purification. NMR spectra were recorded with a *BRUKER Avance 400* and a *Bruker Avance 600* at 400/600 MHz (^{1}H NMR) and 100/151 MHz (^{13}C NMR). Chemical shifts for ^{1}H NMR spectra were reported in ppm relative to the signal of chloroform-d (δ 7.26).[1] Chemical shifts for ^{13}C NMR spectra were reported in ppm relative to the signal of chloroform-d (δ 77.00).[1] Mass spectra with high resolution were recorded on a maXis-4G-spectrometer from *Bruker Daltonics* (ESI, APCI or EI) with a TOF analyzer.

Optical measurements

UV/Vis spectra have been measured at rt and 1 x 10^{-4} M in DCM on a Lambda 1050 spectrometer from *PerkinElmer* with a *PerkinElmer* 3D WB Det module.

Fluorescence emission spectra have been taken on a PTI Quantamaster QM4 Spektrofluorimeter from *Horiba* in DCM at rt.

Cyclic voltammetry (CV)

CV experiments were performed in a homebuilt electrochemistry cell which was placed into a Faraday cage within a nitrogen-filled glovebox. The cell involves a Pt coil (30 cm length, 3 mm diameter) as counter electrode, a Glassy Carbon electrode (Metrohm 6.09395.014) is used as working electrode and an Ag/Ag^+ Haber-Luggin double-reference system[2] is employed as reference electrode. The cell involves a Pt coil (30 cm length, 3 mm diameter) as counter electrode, a Glassy Carbon (GC) electrode (Metrohm 6.09395.014) is used as working electrode and an Ag/Ag+ Haber-Luggin double-reference system is employed as reference electrode. Before use, the GC electrode was cleaned by neutral 0.3 µm aluminium oxide on a polishing pad. The reference consists of an Ag wire which is placed into 2 ml 0.01 M $AgClO_4$ (≥97%, anhydrous, Alfa Aesar) in a 0.1M solution of tetrabutylammonium hexafluorophosphate in acetonitrile (CH_3CN/TBAHFP). The next compartment, separated by a frit, is filled with 5 ml of 0.1 M CH_3CN/TBAHFP and again separated by a frit 7 ml of the used electrolyte solution 0.1 M THF/TBAHFP.

A CHI760E Bipotentiostat (CH-Instruments) was used, controlled by the CHI760E software. After the addition of the electrolyte solution 12 ml of 0.1M THF/TBAHFP (THF: HPLC-grade, TBAHFP: 98 %, Alfa Aesar, five times recrystallized), all three electrodes were connected to the potentiostat. At first, the open circuit potential was determined and the *iR* compensation was manually applied by the potentiostat. All of the recorded data were referenced by the Ferrocen/Ferrocenium couple[3] within the same electrochemical cell and the same electrolyte solution. This way, a half-wave potential of 184 mV +/- 6 mV vs. Ag/Ag+. was determined. For each measurement, the background (electrolyte solution) was recorded and stock solutions of the compounds of approx. 10 mM were prepared. For the compound measurements, 100 µl of the prepared stock solutions were added.

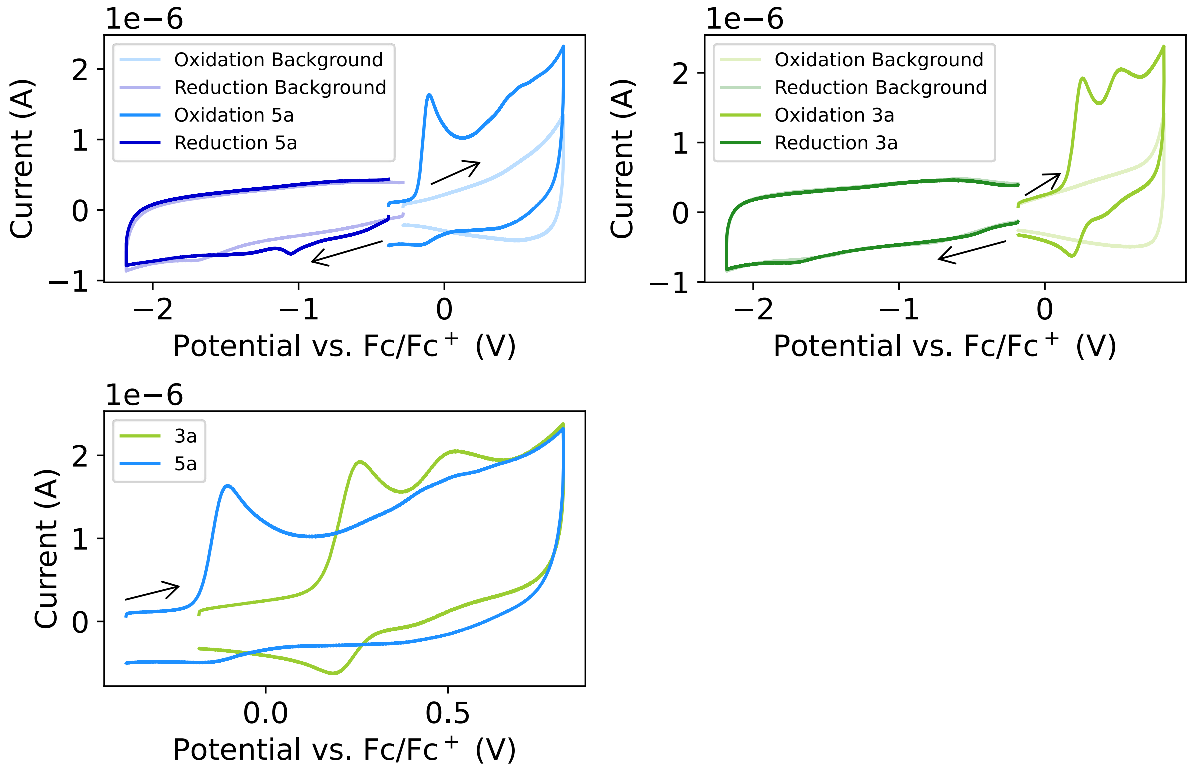

Figure S1. Cyclic Voltammetry in oxidative and reductive scan direction with background (electrolyte solution, light lines) at a scan speed of 100 mV/s in THF/TBAHFP (0.1 M) a) of compound **5a** and b) of compound **3a**. c) Comparison of the oxidative scan directions of the two compounds. All measured data is plotted according to IUPAC convention.

Both compounds show no reduction within the electrochemical window up to approx. -2 V vs. Fc/Fc$^+$. In the oxidative direction, compound 5a shows a partially reversible oxidation with a half-wave potential of -0.14 V vs. Fc/Fc$^+$. Compound 3a can be oxidized partially reversible at 0.23 V vs. Fc/Fc$^+$. It shows also a second irreversible oxidation at higher potentials at approx. 0.4 V vs. Fc/Fc$^+$.

Thin Film Fabrication

For electrical measurements, a thin film of compound **5a** was prepared on commercially available OFET substrates (Fraunhofer IPMS, Dresden), consisting of interdigitated Au electrodes with a channel length of L = 2.5 µm and a channel width of W = 1 cm on a Si wafer with 200 nm SiO_X layer. The substrates (15x15 mm^2) were coated with 100 µl of a solution of compound **5a** (0.5 wt. % in dichloromethane) and spincoated at 900 rpm for 1 min. Spincoating was performed under ambient conditions in a fume hood. Through profilometry (Bruker, Dektak XT-A), a film thickness of h ≈ 60 nm was determined.

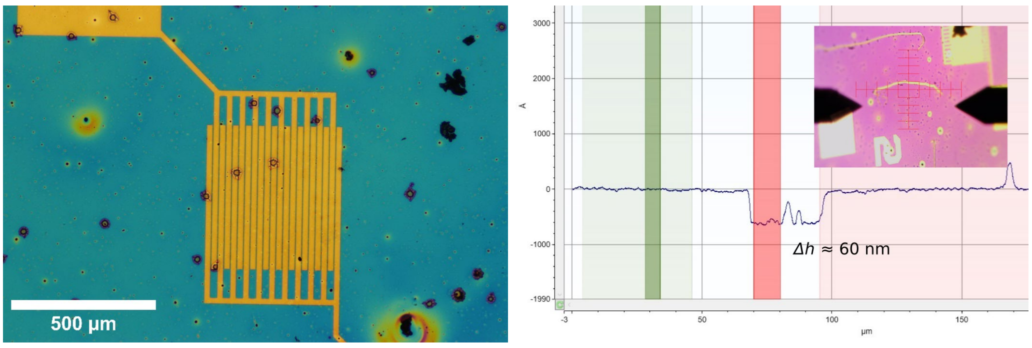

Figure S2. Left: Optical micrograph of a thin film of compound **5a**, spincoated on an OFET electrode structure (device A1). Right: Profilometry of the thin film across an intentional scratch.

Dark and Photocurrent measurements

Electrical measurements were executed at room temperature under vacuum (3×10^{-5} mbar) in a Lake Shore Cryotronics probe station CRX-6.5K with a Keithley 2636B System Source Meter, and the samples were contacted with tungsten two-point probes. For photocurrent measurements, a single-mode fiber-pigtailed laser diode (Thorlabs, S405-XP) with λ = 408.7 nm and an optical output power of 150 µW was operated with a laser diode controller (Thorlabs, LDC210C) using the continuous wave mode and manually switching with a laser pulse duration of 8 s while a constant bias voltage of 20 V was applied.

Computational Methods

DFT geometry and energy calculations were performed using M06-2X/6-311+G** SMD=THF on *Gaussian*[4] with the help of *Gauss View*.[5] TS3b in Figure 3 was calculated using UM06-2X/6-311+G** SMD=THF.

HOMO/LUMO energies were calculated using B3LYP/6-311+G**, SMD=THF.

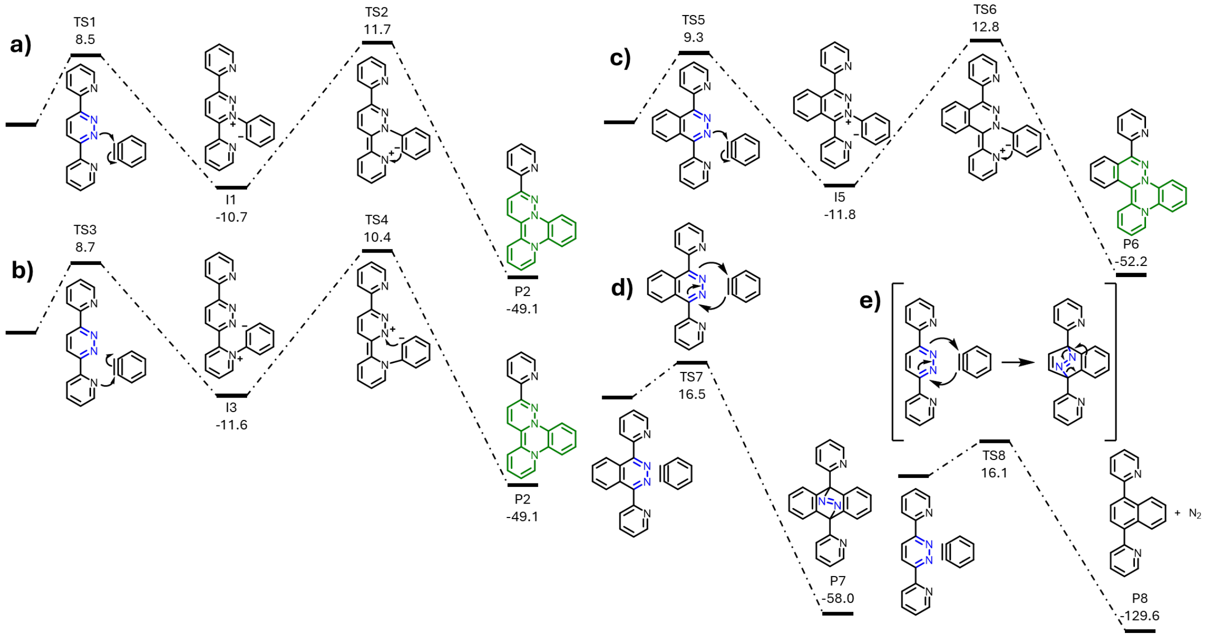

Figure S3. Relative Gibbs energies (calculated at 298 K, in kcal/mol) computed (M06-2X/6-311+G**, SMD=THF) for formation of various products that were not isolated. a) formation of triphenylene P2 by nucleophilic attack of pyridazine nitrogen b) formation of triphenylene P2 by nucleophilic attack of pyridyl nitrogen c) formation of triphenylene P6 by nucleophilic attack of phthalazine nitrogen d) Diels-Alder reaction of phthalazine with benzyne e) Diels-Alder reaction of pyridazine with benzyne.

Crystallographic data

5a

A clear red plate-shaped-shaped crystal with dimensions 0.12 × 0.09 × 0.01 mm^3 was mounted. Data were collected using a XtaLAB Synergy, Dualflex, HyPix diffractometer operating at T = 149.99(10) K.

Formula	$C_{21}H_{16}Cl_2N_4$
CCDC	2288131
$D_{calc.}$/ g cm^{-3}	1.420
μ/mm^{-1}	3.259
Formula Weight	395.293
Colour	clear red
Shape	plate-shaped
Size/mm^3	0.12×0.09×0.01
T/K	149.99(10)
Crystal System	monoclinic
Space Group	$P2_1/c$
a/Å	9.9541(3)
b/Å	17.8265(7)
c/Å	10.5237(4)
α/°	90
β/°	97.927(3)
γ/°	90
V/Å^3	1849.55(12)
Z	4
Z'	1
Wavelength/Å	1.54184
Radiation type	Cu K□
Θ_{min}/°	4.48
Θ_{max}/°	66.58
Measured Refl's.	15083
Indep't Refl's	3209
Refl's I≥2 σ(I)	2591
R_{int}	0.0291
Parameters	393
Restraints	6
Largest Peak	0.4168
Deepest Hole	-0.2349
GooF	1.0528
wR_2 (all data)	0.0693
wR_2	0.0650
R_1 (all data)	0.0421
R_1	0.0301

5c

A clear colourless needle-shaped-shaped crystal with dimensions 0.21 × 0.03 × 0.02 mm^3 was mounted. Data were collected using a XtaLAB Synergy, Dualflex, HyPix diffractometer operating at T = 150.00(10) K.

Formula	$C_{20}H_{12}F_2N_4$
CCDC	2353718
$D_{calc.}$/ g cm^{-3}	1.461
μ/mm^{-1}	0.888
Formula Weight	346.342
Colour	clear colourless
Shape	needle-shaped
Size/mm^3	0.21×0.03×0.02
T/K	150.00(10)
Crystal System	monoclinic
Flack Parameter	-0.03(4)
Hooft Parameter	-0.03(4)
Space Group	$P2_1$
a/Å	8.8074(3)
b/Å	5.8185(2)
c/Å	15.5180(4)
α/°	90
β/°	98.149(3)
γ/°	90
V/$Å^3$	787.20(4)
Z	2
Z'	1
Wavelength/Å	1.54184
Radiation type	Cu K□
Θ_{min}/°	2.88
Θ_{max}/°	80.47
Measured Refl's.	26933
Indep't Refl's	3403
Refl's I≥2 σ(I)	3202
R_{int}	0.0352
Parameters	343
Restraints	1
Largest Peak	0.1069
Deepest Hole	-0.1126
GooF	1.0875
wR_2 (all data)	0.0446
wR_2	0.0438
R_1 (all data)	0.0218
R_1	0.0193

REFERENCES

(1) Fulmer, G. R.; Miller, A. J. M.; Sherden, N. H.; Gottlieb, H. E.; Nudelman, A.; Stoltz, B. M.; Bercaw, J. E.; Goldberg, K. I. NMR Chemical Shifts of Trace Impurities: Common Laboratory Solvents, Organics, and Gases in Deuterated Solvents Relevant to the Organometallic Chemist. *Organometallics* **2010**, *29* (9), 2176-2179.
(2) Bott, A. Practical problems in voltammetry 3: reference electrodes for voltammetry. *Curr. Sep.* **1995**, *14*, 64-69.
(3) Gritzner, G.; Kuta, J. Recommendations on reporting electrode potentials in nonaqueous solvents (Recommendations 1983). *Pure Appl. Chem.* **1984**, *56* (4), 461-466.
(4) *Gaussian 16 Rev. C.01*, Frisch, M. J.; Trucks, G. W.; Schlegel, H. B.; Scuseria, G. E.; Robb, M. A.; Cheeseman, J. R.; Scalmani, G.; Barone, V.; Petersson, G. A.; Nakatsuji, H.; Li, X.; Caricato, M.; Marenich, A. V.; Bloino, J.; Janesko, B. G.; Gomperts, R.; Mennucci, B.; Hratchian, H. P.; Ortiz, J. V.; Izmaylov, A. F.; Sonnenberg, J. L.; Williams; Ding, F.; Lipparini, F.; Egidi, F.; Goings, J.; Peng, B.; Petrone, A.; Henderson, T.; Ranasinghe, D.; Zakrzewski, V. G.; Gao, J.; Rega, N.; Zheng, G.; Liang, W.; Hada, M.; Ehara, M.; Toyota, K.; Fukuda, R.; Hasegawa, J.; Ishida, M.; Nakajima, T.; Honda, Y.; Kitao, O.; Nakai, H.; Vreven, T.; Throssell, K.; Montgomery Jr., J. A.; Peralta, J. E.; Ogliaro, F.; Bearpark, M. J.; Heyd, J. J.; Brothers, E. N.; Kudin, K. N.; Staroverov, V. N.; Keith, T. A.; Kobayashi, R.; Normand, J.; Raghavachari, K.; Rendell, A. P.; Burant, J. C.; Iyengar, S. S.; Tomasi, J.; Cossi, M.; Millam, J. M.; Klene, M.; Adamo, C.; Cammi, R.; Ochterski, J. W.; Martin, R. L.; Morokuma, K.; Farkas, O.; Foresman, J. B.; Fox, D. J., Wallingford, CT, 2016.
(5) *Gauss View 6.1*, Dennington, R. K., T. A., Millam, J. M., Semichem Inc., Shawnee Mission, KS,, 2016.

NMR

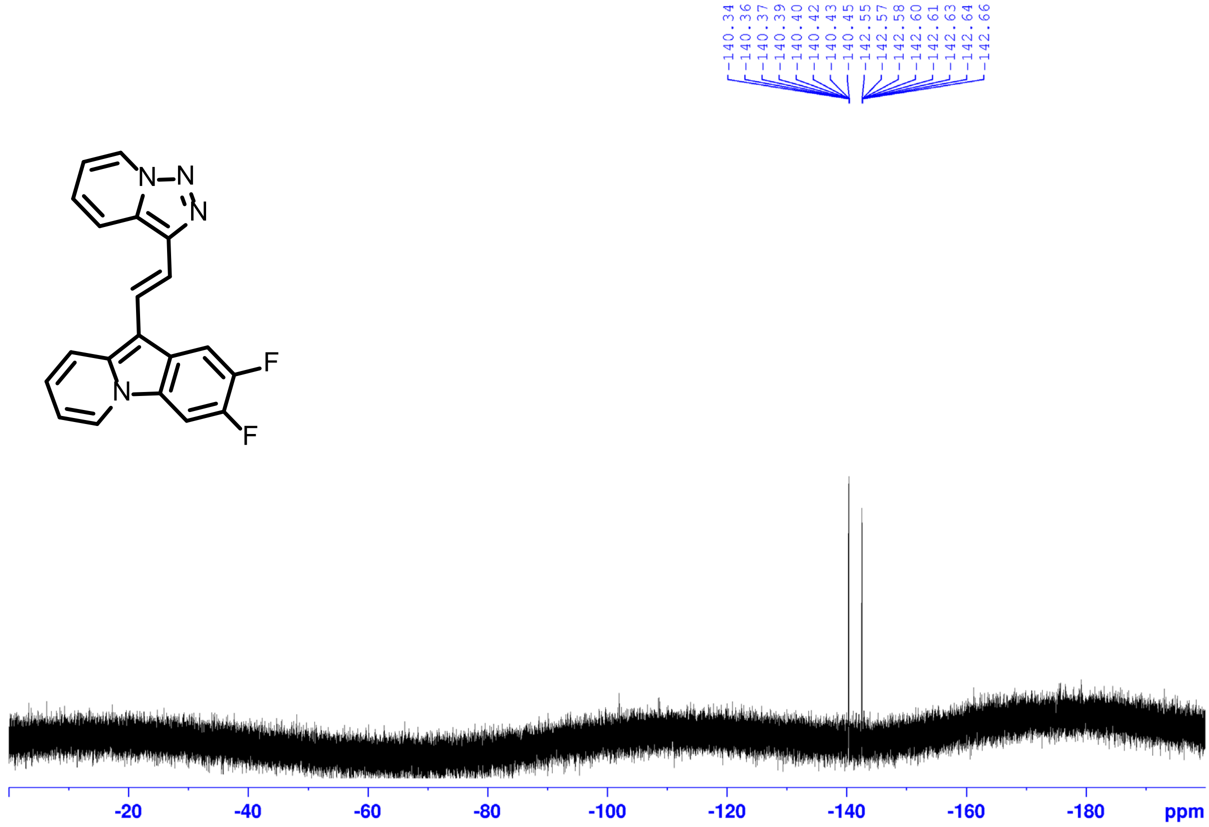

Figure 1 ^{19}F NMR (377 MHz) of **5c** ($CDCl_3$).

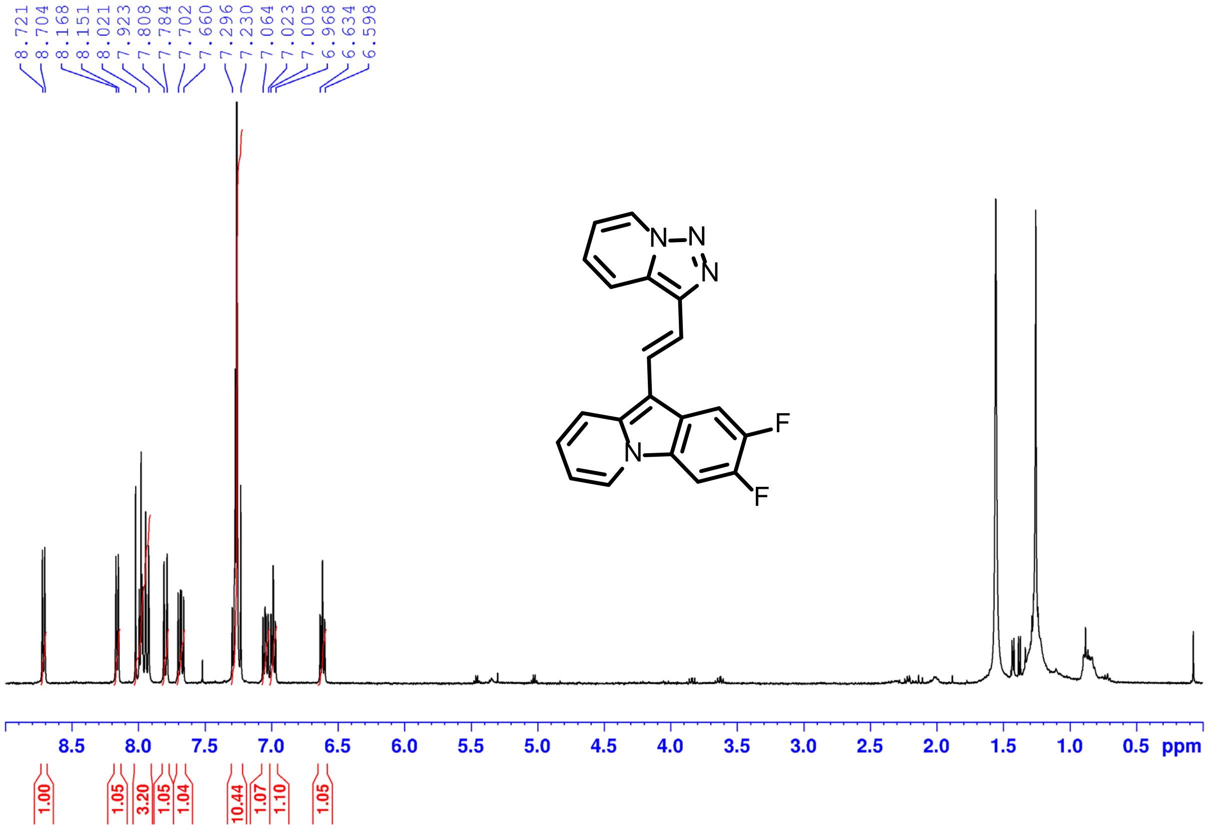

Figure 2 ^{1}H NMR (400 MHz) of **5c** ($CDCl_3$).

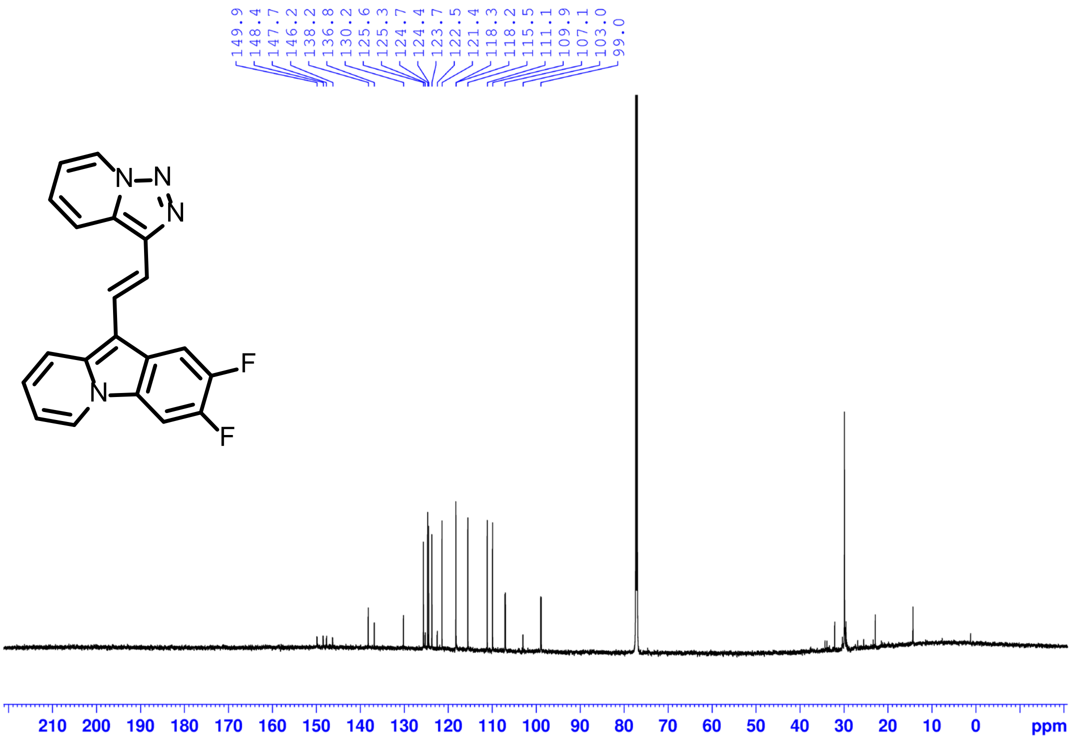

Figure 3 ^{13}C NMR{^{1}H} (176 MHz) of **5c** (CDCl₃).

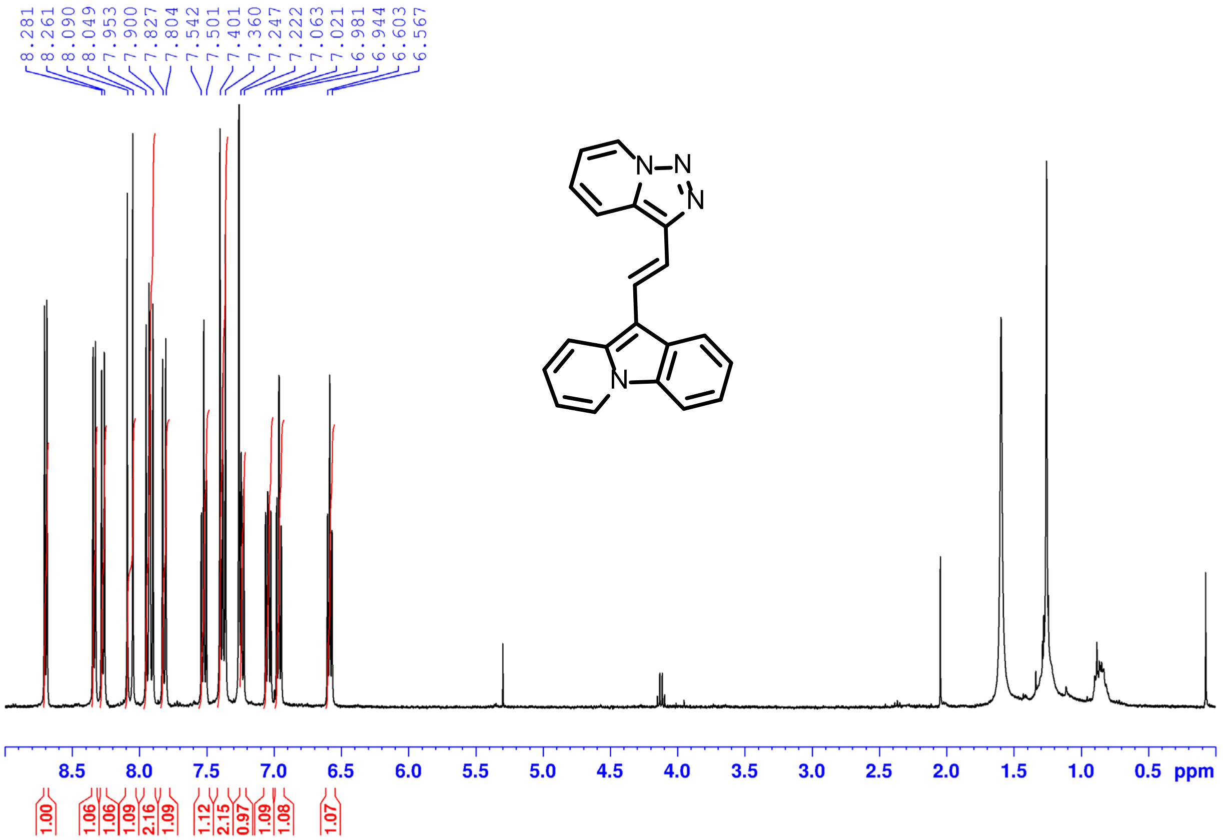

Figure 4 ^{1}H NMR (400 MHz) of **5a** (CDCl₃).

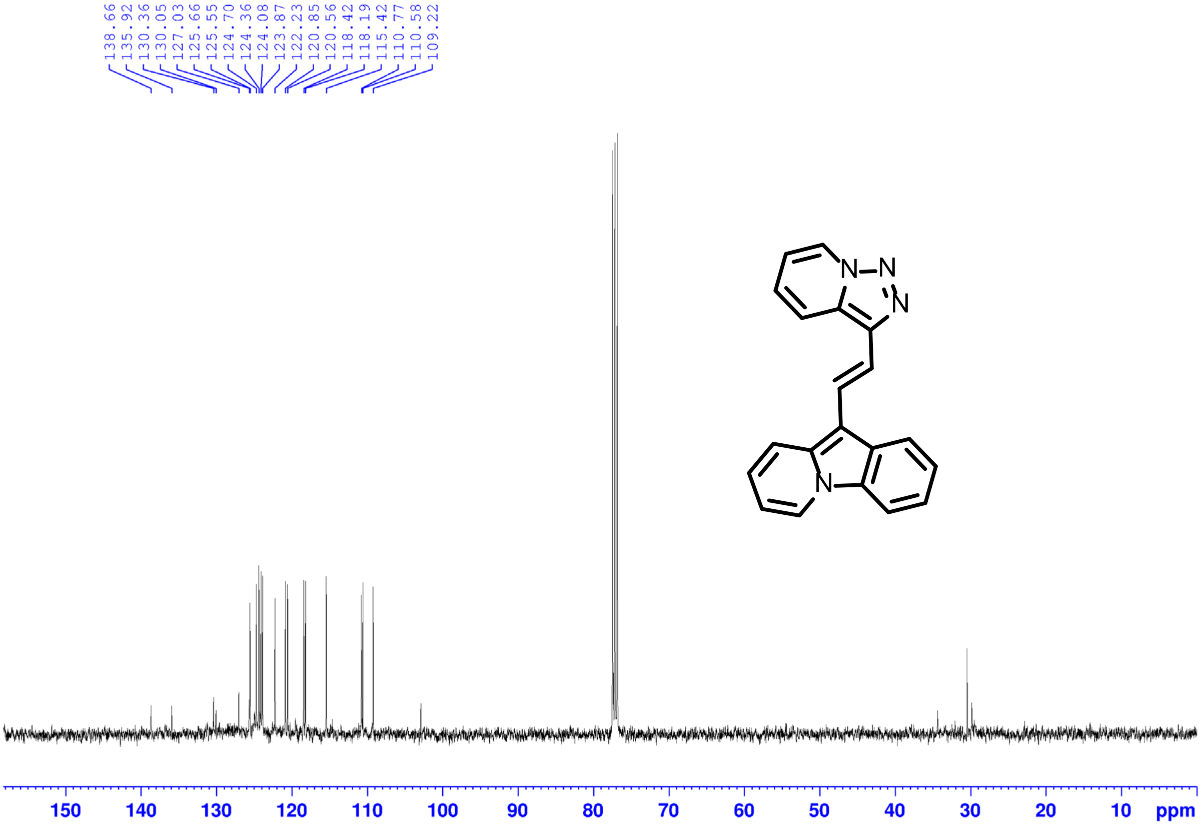

Figure 5 ^{13}C NMR{^{1}H} (176 MHz) of **5a** ($CDCl_3$).

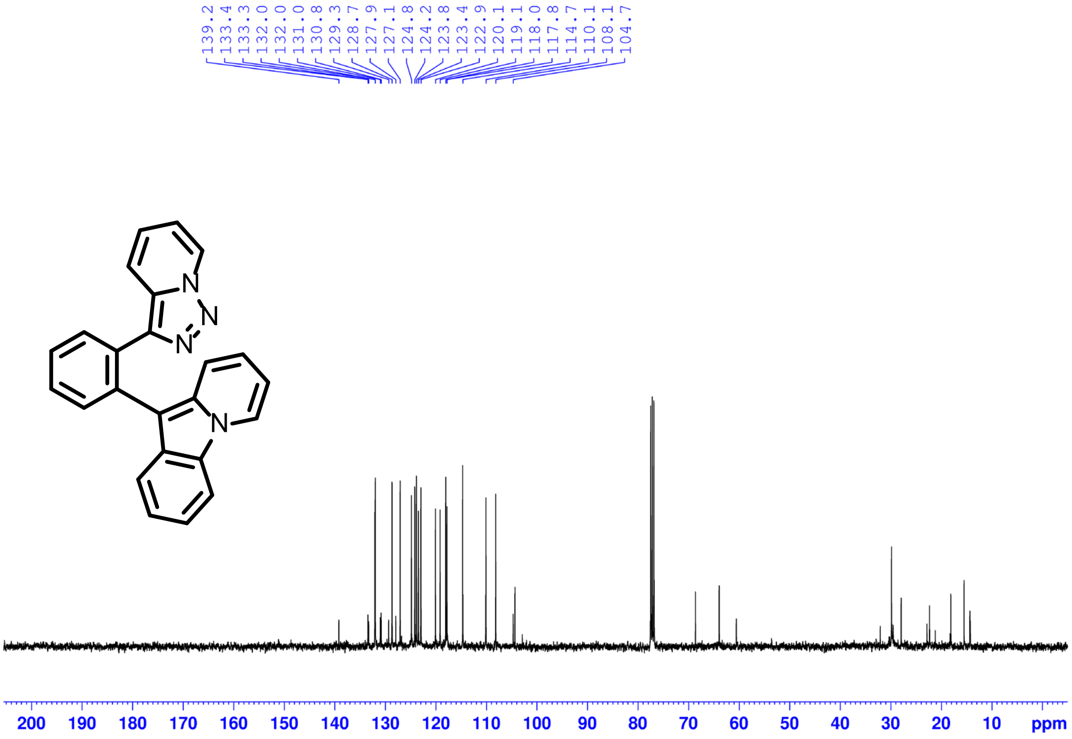

Figure 6 ^{13}C NMR{^{1}H} (176 MHz) of **3a** ($CDCl_3$).

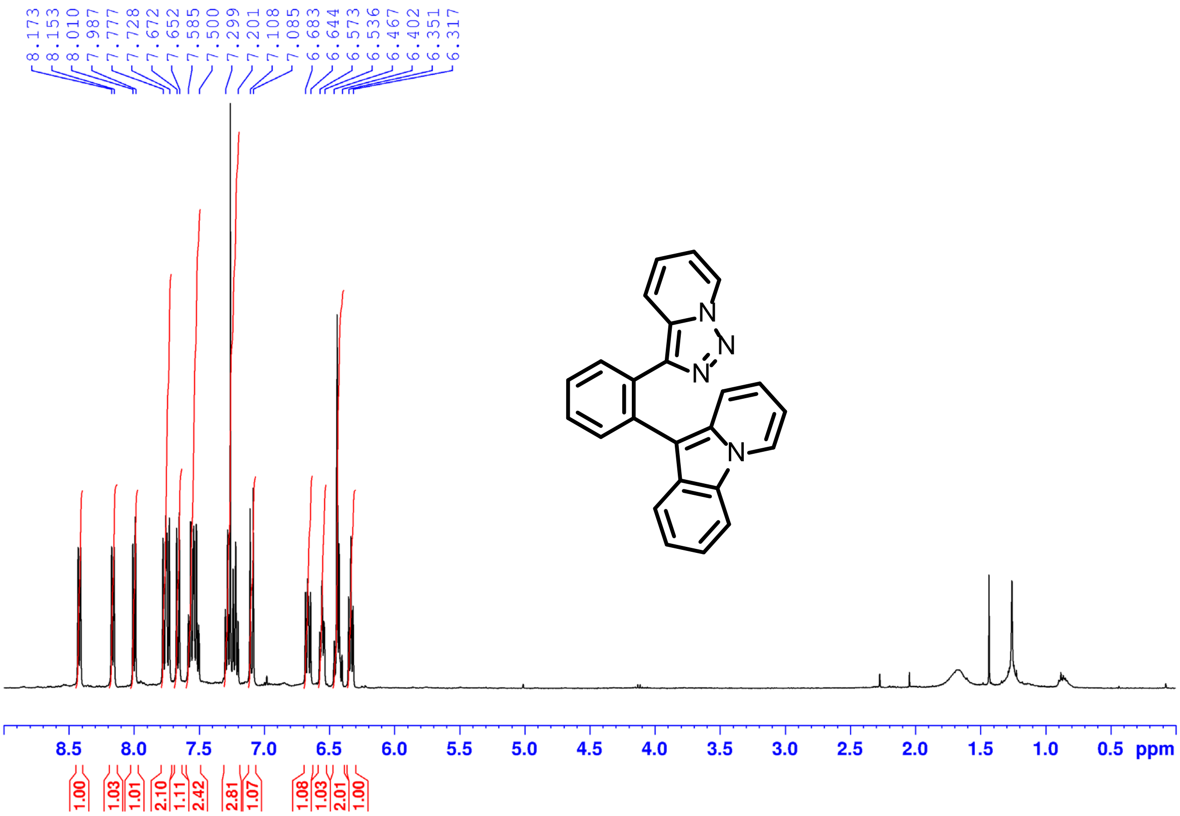

Figure 7 ^{1}H NMR (400 MHz) of **3a** ($CDCl_3$).

Mass spectrometry

Analysis Info

Analysis Name	D:\Data\oi\Bettinger_Schoentag_JS183.1_GE2_01_62663.d	Acquisition Date	3/16/2023 11:05:08 AM
Method	fia_ms_80-1000_pos_neu.m	Operator	BDAL@DE
Sample Name	Bettinger_Schoentag_JS183.1	Instrument	maXis 288882.21253
Comment			

Acquisition Parameter

Source Type	ESI	Ion Polarity	Positive	Set Nebulizer	1.2 Bar
Focus	Not active	Set Capillary	4500 V	Set Dry Heater	200 °C
Scan Begin	80 m/z	Set End Plate Offset	-500 V	Set Dry Gas	6.0 l/min
Scan End	1000 m/z	Set Charging Voltage	0 V	Set Divert Valve	Waste
		Set Corona	0 nA	Set APCI Heater	0 °C

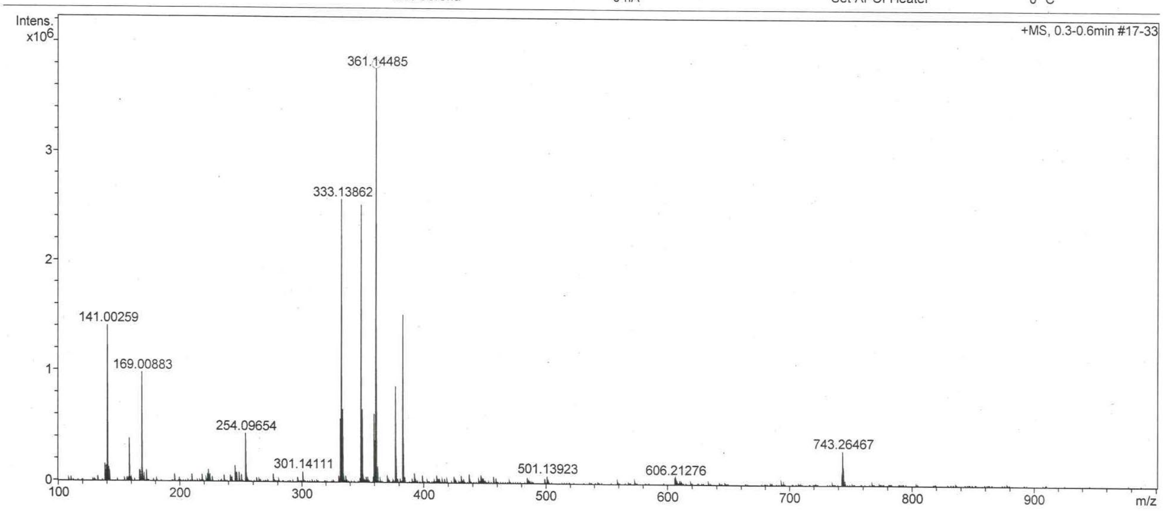

	Meas. m/z	#	Ion Formula	m/z	err [ppm]	mSigma	# mSigma	Score	rdb	e⁻ Conf	N-Rule
[M+H]+	361.14485	1	C24H17N4	361.14477	-0.22	1.5	1	100.00	18.5	even	ok

Figure 8 ESI-HRMS spectrum of **3a**.

Analysis Info

Analysis Name	D:\Data\oi\Bettinger_Schoentag_JS261.2_GE3_01_62659.d	Acquisition Date	3/16/2023 10:26:19 AM
Method	fia_ms_80-1000_pos_neu.m	Operator	BDAL@DE
Sample Name	Bettinger_Schoentag_JS261.2	Instrument	maXis 288882.21253
Comment			

Acquisition Parameter

Source Type	ESI	Ion Polarity	Positive	Set Nebulizer	1.2 Bar
Focus	Not active	Set Capillary	4500 V	Set Dry Heater	200 °C
Scan Begin	80 m/z	Set End Plate Offset	-500 V	Set Dry Gas	6.0 l/min
Scan End	1000 m/z	Set Charging Voltage	0 V	Set Divert Valve	Waste
		Set Corona	0 nA	Set APCI Heater	0 °C

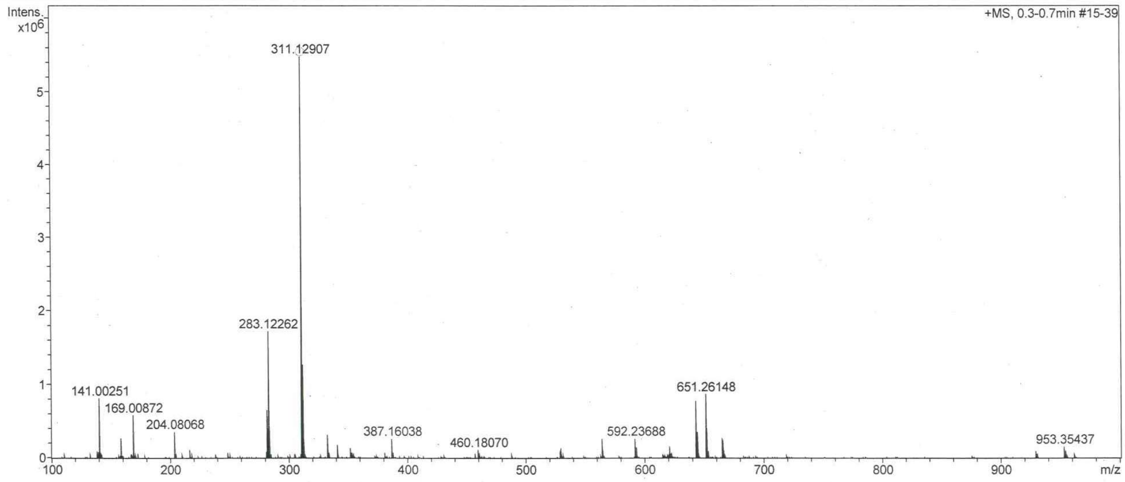

	Meas. m/z	#	Ion Formula	m/z	err [ppm]	mSigma	# mSigma	Score	rdb	e⁻ Conf	N-Rule
[M+H]+	311.12907	1	C20H15N4	311.12912	0.16	12.5	1	100.00	15.5	even	ok

Figure 9 ESI-HRMS spectrum of **5a**.

Acquisition Parameter

Source Type	ESI	Ion Polarity	Positive	Set Nebulizer	1.2 Bar
Focus	Not active	Set Capillary	4500 V	Set Dry Heater	200 °C
Scan Begin	80 m/z	Set End Plate Offset	-500 V	Set Dry Gas	6.0 l/min
Scan End	1000 m/z	Set Charging Voltage	0 V	Set Divert Valve	Waste
		Set Corona	0 nA	Set APCI Heater	0 °C

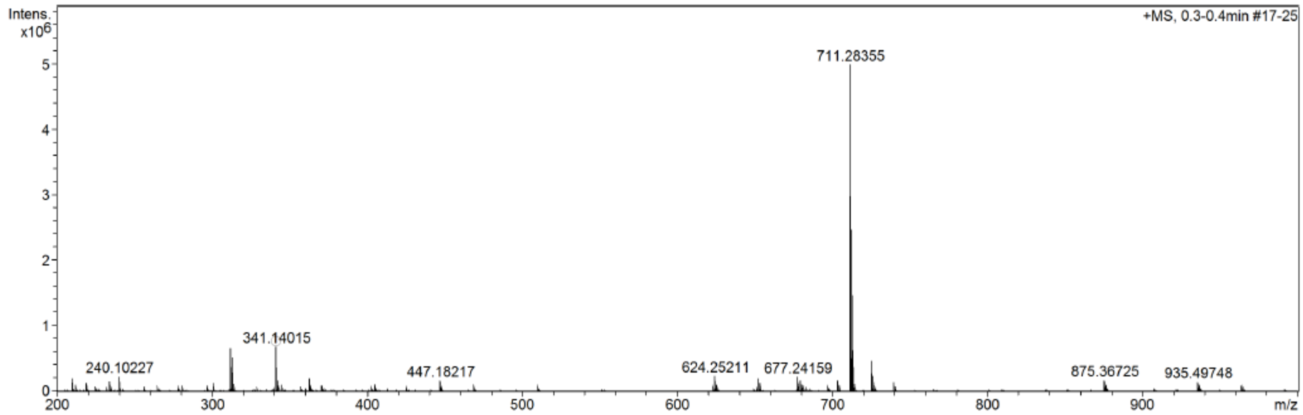

	Meas. m/z	#	Ion Formula	m/z	err [ppm]	mSigma	# mSigma	Score	rdb	e⁻ Conf	N-Rule
$[M+H]^+$	341.14015	1	C21H17N4O	341.13969	-1.37	25.8	1	100.00	15.5	even	ok

Figure 10 ESI-HRMS spectrum of **5b**.

Acquisition Parameter

Source Type	ESI	Ion Polarity	Positive	Set Nebulizer	1.2 Bar
Focus	Not active	Set Capillary	4500 V	Set Dry Heater	200 °C
Scan Begin	80 m/z	Set End Plate Offset	-500 V	Set Dry Gas	6.0 l/min
Scan End	1000 m/z	Set Charging Voltage	0 V	Set Divert Valve	Waste
		Set Corona	0 nA	Set APCI Heater	0 °C

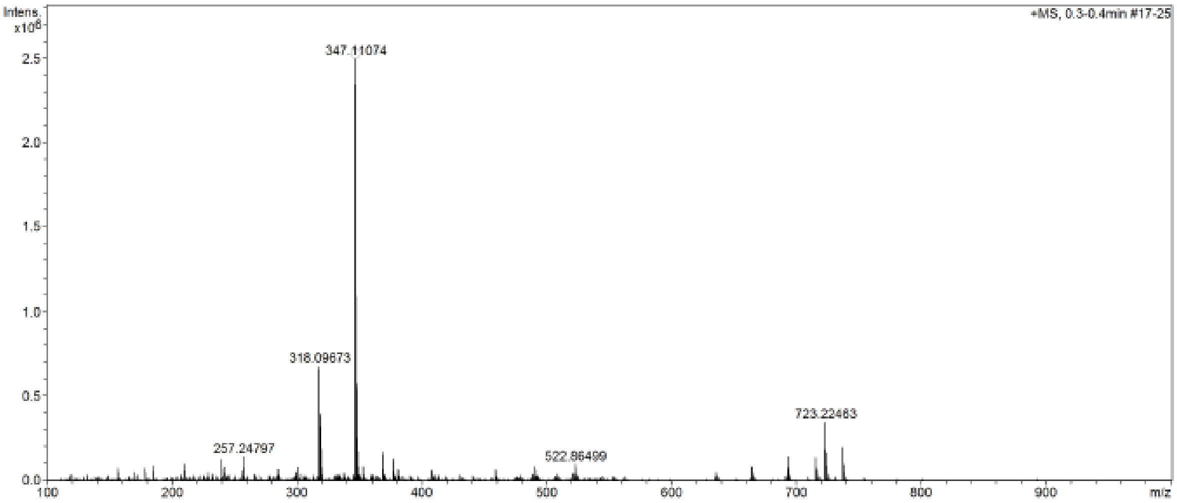

	Meas. m/z	#	Ion Formula	m/z	err [ppm]	mSigma	# mSigma	Score	rdb	e⁻ Conf	N-Rule
$[M+H]^+$	347.11074	1	C20H13F2N4	347.11028	-1.33	27.6	1	100.00	15.5	even	ok

Figure 11 ESI-HRMS spectrum of **5c**.

Computational data

Figure 2

TS1

C	-4.85553600	-1.00661500	-1.00813000
C	-5.41016300	-0.40395400	0.11237200
C	-4.56797900	-0.06634900	1.16978200
N	-3.25732800	-0.29394300	1.16139200
C	-2.73717000	-0.88216700	0.08192900
C	-3.48817300	-1.25254400	-1.03144800
H	-5.47485500	-1.28415500	-1.85289700
H	-6.47037800	-0.19261800	0.17363100
H	-4.96585700	0.41348300	2.05823500
H	-3.01179500	-1.72253600	-1.88143600
N	0.64524600	-1.21330000	1.30444900
C	1.26845500	-1.10448300	0.11050900
C	-1.26851200	-1.10446100	0.11052300
N	-0.64530500	-1.21329000	1.30445600
C	4.85551200	-1.00680900	-1.00805100
C	3.48814700	-1.25272500	-1.03138300
C	2.73711400	-0.88220400	0.08192600
N	3.25724400	-0.29385700	1.16133600
C	4.56789700	-0.06627700	1.16974000
C	5.41011000	-0.40401900	0.11239600
H	5.47485600	-1.28446200	-1.85276200
H	3.01178900	-1.72281700	-1.88132600
H	4.96575300	0.41365600	2.05814900
H	6.47032500	-0.19268800	0.17366400
N	0.64619200	-1.44555700	-1.03372500
N	-0.64625400	-1.44554500	-1.03371700
C	-0.62424300	1.36362500	-0.12780300
C	0.62428400	1.36357200	-0.12780400
C	-1.46164000	2.45700700	-0.24172700
H	-2.54414600	2.45031700	-0.23375200
C	1.46179800	2.45685800	-0.24174400
C	-0.70234500	3.63352500	-0.36914600

H	2.54430300	2.45005500	-0.23377300
H	-1.22562100	4.57886200	-0.46687100
C	0.70262200	3.63345400	-0.36915700
H	1.22599500	4.57873700	-0.46688900

2

C	-4.82059500	-1.73162500	-0.54042600
C	-5.52288100	-0.87110800	0.29429200
C	-4.81462500	0.11258900	0.97604600
N	-3.49589900	0.27654200	0.86634900
C	-2.82663200	-0.55920200	0.06676900
C	-3.44590400	-1.57936400	-0.65564200
H	-5.33411800	-2.51029700	-1.09234500
H	-6.59520300	-0.95469100	0.41935900
H	-5.32813300	0.79956300	1.64132300
H	-2.85403800	-2.22871100	-1.28758000
C	1.34439800	-0.39615300	-0.00276800
C	-1.34441200	-0.39613000	-0.00277500
C	4.82057600	-1.73167400	-0.54038500
C	3.44588400	-1.57942500	-0.65559500
C	2.82661500	-0.55923600	0.06678200
N	3.49588700	0.27655500	0.86630800
C	4.81461500	0.11261400	0.97599800
C	5.52286700	-0.87111500	0.29428600
H	5.33409800	-2.51036900	-1.09227400
H	2.85401500	-2.22880000	-1.28750000
H	5.32812700	0.79962300	1.64123600
H	6.59519000	-0.95468700	0.41934600
N	0.67422400	-1.51965100	0.04502500
N	-0.67425600	-1.51963800	0.04501900
C	-0.70641800	0.88188400	-0.13514300
C	0.70642800	0.88186900	-0.13513700
C	-1.40501500	2.09680300	-0.33149000
H	-2.48555800	2.09952500	-0.34513200
C	1.40505900	2.09677200	-0.33146400

C	-0.70514400	3.26343100	-0.51283900
H	2.48560300	2.09946500	-0.34508500
H	-1.23959300	4.19333700	-0.66819600
C	0.70521900	3.26341500	-0.51282400
H	1.23969300	4.19330900	-0.66817000

TS2

C	-3.51147000	3.14980600	-1.35740600
C	-4.50898200	2.19527600	-1.20887200
C	-4.14137500	0.90847800	-0.83067000
N	-2.87903800	0.54490300	-0.61944700
C	-1.92130800	1.46554900	-0.75816800
C	-2.19274000	2.78384300	-1.11927200
H	-3.75346700	4.16409400	-1.65250900
H	-5.55110300	2.43502600	-1.37816200
H	-4.88910600	0.13433800	-0.68078000
H	-1.38256700	3.49643200	-1.22198500
C	1.99422200	0.06692900	-0.38170700
C	-0.51487000	1.01127200	-0.53953200
C	4.76690900	-2.46407900	-0.62305000
C	3.49015400	-1.92796900	-0.52997100
C	3.35576300	-0.54243500	-0.44113900
N	4.39897500	0.29262500	-0.44320800
C	5.61946800	-0.23516500	-0.54343100
C	5.85680900	-1.60244300	-0.63319300
H	4.90801600	-3.53670800	-0.68748200
H	2.60957400	-2.55730600	-0.52430600
H	6.44591600	0.46847000	-0.55317000
H	6.87083700	-1.97466900	-0.70836100
N	1.12899700	-0.46076300	-1.21068100
N	-0.13116700	0.01673700	-1.29485100
C	0.33487700	1.62114900	0.43871700
C	-0.09987500	2.62481700	1.33571800
C	1.65552700	1.12822400	0.52263000
C	0.76901500	3.11865600	2.27645300

C	2.52842800	1.64433500	1.50917200
C	2.08728600	2.62444800	2.36362600
H	-1.11957100	2.98584800	1.29153800
H	0.43730300	3.88304700	2.96918400
H	3.53601200	1.26213700	1.58909300
H	2.75471100	3.01638600	3.12209600
C	-4.05635200	-2.02877600	0.97729900
C	-3.02502300	-1.78733900	0.30616400
C	-4.21568300	-3.34681800	1.43861100
H	-5.06071500	-3.68651200	2.02761400
C	-1.94722700	-2.49948400	-0.14360600
C	-3.19048500	-4.23196900	1.07961700
H	-1.12392200	-2.14801000	-0.74952600
H	-3.24948400	-5.26724400	1.39930600
C	-2.08881600	-3.82377300	0.30997000
H	-1.32230300	-4.54503700	0.04699800

I2

C	-3.97320600	2.20753200	-1.65847400
C	-4.86596800	1.19042000	-1.35214900
C	-4.39360800	0.05059200	-0.73401300
N	-3.09082100	-0.09257500	-0.42983700
C	-2.19628500	0.86987700	-0.75385500
C	-2.62842500	2.04069900	-1.35501400
H	-4.31428100	3.11625500	-2.13910400
H	-5.92118800	1.27018100	-1.57523000
H	-5.02349800	-0.78103500	-0.45201800
H	-1.89510700	2.79606100	-1.60760500
C	1.87798200	0.08250300	-0.46098200
C	-0.73553600	0.62694300	-0.55241400
C	4.97987100	-2.02488800	-0.72878200
C	3.64100300	-1.67688300	-0.61416300
C	3.31136200	-0.32357000	-0.54535700
N	4.22170300	0.65379000	-0.58765000
C	5.50291900	0.30536700	-0.70931400

C	5.93294400	-1.01542800	-0.78067600
H	5.27252700	-3.06716400	-0.77840100
H	2.86036800	-2.42572000	-0.57608500
H	6.21844300	1.12044100	-0.75253500
H	6.98827600	-1.23912600	-0.87432900
N	1.08081200	-0.58886800	-1.25659900
N	-0.23815500	-0.31838000	-1.30035300
C	0.03469100	1.41346100	0.35598900
C	-0.52016900	2.39780500	1.20553400
C	1.41257400	1.11444400	0.42013600
C	0.28991900	3.05468900	2.09728300
C	2.22312500	1.79503800	1.35931800
C	1.66475000	2.74557500	2.17771200
H	-1.57986300	2.61966600	1.15993200
H	-0.12832500	3.80688100	2.75564100
H	3.27578200	1.56190600	1.43039400
H	2.28462600	3.26254200	2.90068200
C	-3.06008100	-2.54275300	-0.32983900
C	-2.69551500	-1.34403400	0.26974000
C	-2.62484800	-3.65071400	0.43563900
H	-2.83965700	-4.65638100	0.07579600
C	-2.02856600	-1.16113400	1.48297100
C	-1.92442700	-3.55000500	1.63828300
H	-1.84161700	-0.17192300	1.89118500
H	-1.61492500	-4.44620100	2.16835700
C	-1.63147700	-2.29591600	2.17726900
H	-1.11450800	-2.20187700	3.12532400

TS3

C	4.06135500	2.11189700	-1.49927000
C	4.94809300	1.09208600	-1.17685600
C	4.46240400	-0.05080500	-0.57896100
N	3.14885900	-0.19614800	-0.32011400
C	2.26408500	0.78172300	-0.62384800
C	2.71223100	1.95066500	-1.21584000

H	4.41535200	3.02226900	-1.96719700
H	6.00944000	1.17759500	-1.36574400
H	5.10345600	-0.86167400	-0.26393500
H	1.98777300	2.71584000	-1.46442500
C	-1.84096900	0.14564600	-0.51010300
C	0.80519600	0.53030100	-0.40645700
C	-5.15490000	-1.10294200	-1.78980700
C	-3.84099300	-0.65899700	-1.78142900
C	-3.26464800	-0.29274100	-0.56235700
N	-3.93098300	-0.34909500	0.59585500
C	-5.19177300	-0.78317200	0.57426500
C	-5.84947100	-1.17066300	-0.58734200
H	-5.63051700	-1.38983600	-2.72067100
H	-3.25977200	-0.59219100	-2.69163600
H	-5.69911900	-0.82504300	1.53316600
H	-6.87561200	-1.51370700	-0.54445800
N	-1.04043700	-0.48465000	-1.32885800
N	0.28376700	-0.23688600	-1.35022900
C	0.00271600	1.43214200	0.38453900
C	0.53848800	2.45468500	1.18835000
C	-1.38604700	1.22681000	0.33398200
C	-0.30557600	3.28179700	1.89445400
C	-2.23669900	2.09702000	1.05007000
C	-1.69964100	3.10985400	1.81234900
H	1.61270200	2.58080400	1.26009800
H	0.10354900	4.06887000	2.51718400
H	-3.30809800	1.96208800	1.00109000
H	-2.35521100	3.77539300	2.36172900
C	1.51032300	-1.14057500	1.14937500
C	2.62947000	-1.38384800	0.37091000
C	1.00058800	-2.28622800	1.78181200
H	0.12021700	-2.20726700	2.41684200
C	3.24324600	-2.61550500	0.15611700
C	1.58377200	-3.54700000	1.63109800
H	4.08542800	-2.74282700	-0.51582200

H	1.16063400	-4.40815800	2.13964600
C	2.70230200	-3.71518800	0.81404900
H	3.14002500	-4.69647500	0.67295300

I3

C	-4.17561700	-1.77376600	-1.64984700
C	-5.04003700	-0.74549600	-1.26917300
C	-4.53783900	0.35162900	-0.60718900
N	-3.22029500	0.40326900	-0.33558400
C	-2.35759400	-0.58258200	-0.68035000
C	-2.82063300	-1.69178000	-1.35776200
H	-4.56426300	-2.63650600	-2.17698600
H	-6.09803800	-0.78964100	-1.48779200
H	-5.14018200	1.18899500	-0.28451100
H	-2.11958700	-2.46567600	-1.64303900
C	1.76744700	-0.22124600	-0.50411900
C	-0.94953800	-0.21076900	-0.30767400
C	5.12013800	0.84529100	-1.83797200
C	3.78943200	0.48010300	-1.81302200
C	3.18822200	0.13037100	-0.58401100
N	3.89643500	0.15047000	0.55971300
C	5.18457100	0.50260000	0.51391500
C	5.85045600	0.86119800	-0.64703500
H	5.59361400	1.11001300	-2.77737600
H	3.19881900	0.45455400	-2.71862100
H	5.70865000	0.50098900	1.46602200
H	6.89614300	1.14033700	-0.62100500
N	1.02432500	0.11761200	-1.56434900
N	-0.25287400	0.03579300	-1.62976500
C	-0.21085700	-1.23135800	0.51551100
C	-0.84197400	-2.20354400	1.29189500
C	1.19157900	-1.10439100	0.49741200
C	-0.09375800	-3.05486200	2.09259100
C	1.93343200	-1.98603400	1.31340600
C	1.29878600	-2.93377500	2.09864700

H	-1.92485400	-2.27885200	1.29074100
H	-0.58553900	-3.79512600	2.71264100
H	3.01241400	-1.92255900	1.31660900
H	1.89492600	-3.59404200	2.71918700
C	-1.17505400	1.10995400	0.41114500
C	-2.51764100	1.46040800	0.33825200
C	-0.29443600	1.95977100	1.06799800
H	0.75340400	1.70495100	1.16967400
C	-3.04461800	2.62817700	0.85889800
C	-0.79313900	3.14868600	1.59963800
H	-4.09596400	2.87847300	0.78788800
H	-0.11842700	3.82591900	2.10982000
C	-2.14456100	3.48106000	1.49365400
H	-2.50485900	4.41029800	1.91781400

TS4

C	-4.29363200	1.52960700	1.63028000
C	-5.02320600	0.34143300	1.42113800
C	-4.44596500	-0.69258800	0.73931400
N	-3.18293200	-0.55080800	0.26249900
C	-2.44422300	0.59137900	0.44136400
C	-3.00885300	1.65411300	1.15242600
H	-4.74811200	2.34624900	2.17779700
H	-6.03047100	0.22979800	1.79761000
H	-4.93537300	-1.63822700	0.55015000
H	-2.41976200	2.54731200	1.31603800
C	1.68909100	0.20070200	0.49882500
C	-1.10925600	0.37324500	-0.05448300
C	4.86042300	-1.30771000	1.83042000
C	3.55780100	-0.85238700	1.78866900
C	3.07612100	-0.24678500	0.60912700
N	3.86388700	-0.10270600	-0.47000600
C	5.12330200	-0.54320500	-0.40645000
C	5.67659600	-1.15386200	0.70808300
H	5.24477800	-1.77263400	2.73173000

H	2.90553500	-0.95053200	2.64705700
H	5.71835500	-0.40187600	-1.30452400
H	6.70408500	-1.49431200	0.69840200
N	0.84779200	-0.24215500	1.44265900
N	-0.34716500	-0.22517300	1.57678100
C	-0.20769100	1.45477600	-0.49471100
C	-0.71116400	2.61445900	-1.09953800
C	1.18686000	1.26963300	-0.35486900
C	0.14054400	3.57859300	-1.60990400
C	2.03631000	2.26179000	-0.89335400
C	1.52292800	3.38535700	-1.51417300
H	-1.78523900	2.73151400	-1.20083300
H	-0.26064300	4.46057000	-2.09519500
H	3.10693000	2.13993300	-0.80701600
H	2.20289800	4.12520700	-1.92206600
C	-1.17402000	-0.92353300	-0.73455000
C	-2.42474300	-1.49531000	-0.47540700
C	-0.24998600	-1.63343100	-1.50259700
H	0.71636100	-1.20472400	-1.74136800
C	-2.79845200	-2.75384000	-0.92227600
C	-0.60557900	-2.89706800	-1.95901300
H	-3.77473900	-3.17359400	-0.71298400
H	0.09824100	-3.46345700	-2.55766600
C	-1.85812200	-3.45433700	-1.66840500
H	-2.10443900	-4.44152300	-2.04015800

I4

C	4.63093900	-1.05056100	1.05837100
C	5.08241500	0.30152800	0.91657300
C	4.25484100	1.23362900	0.38646200
N	2.99391200	0.87660300	-0.02059200
C	2.51478800	-0.43895800	0.07577500
C	3.38431700	-1.41208300	0.65146100
H	5.29325200	-1.78651300	1.49805000
H	6.07461200	0.59174800	1.23337500

H	4.52167500	2.27419400	0.25834000
H	3.02424500	-2.42795600	0.75714600
C	-1.48203200	-0.51638200	0.81499500
C	1.21380200	-0.48791200	-0.40511200
C	-4.48864800	1.69634400	1.24731300
C	-3.26079200	1.11327500	1.50412000
C	-2.79258900	0.11594700	0.63192200
N	-3.49892600	-0.29198600	-0.42982100
C	-4.68471600	0.27693500	-0.65437500
C	-5.22705000	1.27444200	0.14391300
H	-4.86935200	2.46960700	1.90484100
H	-2.67683400	1.41978200	2.36393000
H	-5.22503800	-0.08440700	-1.52429500
H	-6.19295900	1.70254400	-0.09107400
N	-0.68211700	0.04057300	1.68394100
N	0.02052600	0.52770100	2.41821200
C	0.35353300	-1.68108800	-0.46258600
C	0.82539600	-2.85253500	-1.07272100
C	-0.95390600	-1.67984400	0.06695400
C	0.02541500	-3.97989000	-1.18828900
C	-1.75933800	-2.81648000	-0.07100600
C	-1.27931200	-3.95690200	-0.69704300
H	1.82697300	-2.85373300	-1.48938800
H	0.41072900	-4.86848400	-1.67508200
H	-2.76463300	-2.80014600	0.33266300
H	-1.91626400	-4.82858300	-0.79225000
C	0.87949200	0.84421000	-0.81058900
C	1.99717200	1.67387300	-0.56214100
C	-0.27221200	1.41848800	-1.38500100
H	-1.13806200	0.80430200	-1.60911800
C	2.00265900	3.04098500	-0.84889500
C	-0.27297500	2.77046300	-1.66619000
H	2.86982600	3.65956500	-0.64941900
H	-1.15365500	3.22368500	-2.10713900
C	0.85287200	3.57879400	-1.39767100

H	0.81728000	4.63648500	-1.63020900

TS5

C	0.31993900	-2.58772400	1.77909200
C	-0.77121400	-3.46784300	1.48553400
C	-1.89159100	-2.97715100	0.90304900
N	-1.96804100	-1.64564300	0.58266200
C	-0.91861300	-0.74878600	0.82929100
C	0.24811800	-1.26552400	1.46675600
H	1.20503900	-2.98369500	2.26246800
H	-0.71733000	-4.51970900	1.72990000
H	-2.75910100	-3.57631400	0.66029200
H	1.05583600	-0.58318400	1.70088600
C	1.47190900	0.62049700	-0.74103000
C	-1.27838400	0.51219900	0.37567400
C	4.93270100	-0.23941600	0.47421800
C	3.76438900	0.48237800	0.36906900
C	2.77856100	0.03230500	-0.53683000
N	2.98292000	-1.07414800	-1.26003300
C	4.11690500	-1.77531600	-1.16208900
C	5.12859100	-1.38989000	-0.30648500
H	5.70152000	0.08176600	1.16762100
H	3.59022800	1.36389300	0.97211900
H	4.19406400	-2.65907600	-1.78586700
H	6.04026100	-1.96887300	-0.24245300
N	0.81564500	-0.17424600	-1.58549800
N	1.14198900	-1.15361400	-2.12869900
C	-0.46019200	1.73635700	0.41658500
C	-1.00937400	2.90366500	0.96185100
C	0.85897300	1.78389900	-0.09166200
C	-0.27731800	4.08124600	1.04791600
C	1.58665500	2.97679500	0.00594600
C	1.03278400	4.11458900	0.57819400
H	-2.02185200	2.86841100	1.34996600
H	-0.72498900	4.96542900	1.48692400

H	2.58881800	3.01715500	-0.40715200
H	1.61424600	5.02736500	0.63688600
C	-2.60399800	0.39119500	-0.15200800
C	-3.01101500	-0.95699300	-0.01612100
C	-3.48949000	1.30007500	-0.76495000
H	-3.20327700	2.33728500	-0.89968400
C	-4.25540900	-1.42066600	-0.44747100
C	-4.72161100	0.84680900	-1.19323900
H	-4.54606700	-2.45810600	-0.33031600
H	-5.41058300	1.53819400	-1.66502200
C	-5.10671700	-0.50175700	-1.03389500
H	-6.08200800	-0.82265400	-1.37997100

3a

C	-1.29391500	0.49452600	-0.37666500
C	1.46625100	0.70807600	0.68403800
C	2.73003500	0.18420100	0.40259100
C	3.79404100	0.43022500	-0.50121400
C	3.92280600	-1.70826100	1.32273900
C	4.88280800	-0.39029500	-0.46350600
H	3.71518800	1.25502700	-1.19824900
C	4.94850800	-1.47210100	0.46748500
H	3.87392500	-2.50323900	2.05420000
H	5.70941400	-0.22898400	-1.14411800
H	5.81576300	-2.11779200	0.49706500
C	-2.55699300	0.32482200	0.16996600
C	-3.46648200	1.20374800	0.82989600
C	-4.65704000	0.73013800	1.28573200
H	-3.17980400	2.23991100	0.96015800
C	-4.14239700	-1.49122000	0.50378300
C	-5.00998300	-0.64787000	1.11220700
H	-5.34843900	1.39465900	1.78968800
H	-4.33451700	-2.54406100	0.34659300
H	-5.95792500	-1.02733000	1.46780800
C	-0.88224200	-0.78890700	-0.85730700

C	0.26861300	-1.25059400	-1.52685000
C	-1.91374700	-1.71514500	-0.57789700
C	0.35837900	-2.58751600	-1.86087100
H	1.06659400	-0.56317200	-1.78432500
C	-1.83000200	-3.06792300	-0.91556400
C	-0.67954400	-3.49359300	-1.55385100
H	1.24090000	-2.95186200	-2.37477200
H	-2.63075000	-3.76106000	-0.68572000
H	-0.57558900	-4.53688200	-1.82730100
N	-2.93653400	-1.02084400	0.04834300
N	2.83191400	-0.87830300	1.26616700
C	0.79007400	1.84986900	0.04602100
C	-0.53771700	1.75696600	-0.42235800
N	1.72816200	-0.99711400	2.02206800
N	0.91909000	-0.04193200	1.66106300
C	1.48618000	3.05653000	-0.07970100
C	0.88249700	4.17943000	-0.63189600
C	-0.43824000	4.10393300	-1.06444000
C	-1.13194700	2.90398200	-0.96449800
H	2.50377300	3.11643300	0.29222800
H	1.43514600	5.10815700	-0.71240200
H	-0.92351800	4.97318200	-1.49349900
H	-2.14822300	2.83624400	-1.33788100

Figure 3

TS1

C	4.84644100	-1.85437600	-0.30088000
C	4.08978900	-2.92599100	0.15553100
C	2.71498700	-2.77314400	0.28175200
C	2.14456400	-1.54948900	-0.06586600
C	4.19125100	-0.66684100	-0.60758700
H	5.92069400	-1.92746800	-0.41345900
H	4.56116700	-3.86863800	0.40776300
H	2.09023300	-3.58990300	0.62294300
H	4.74560400	0.20542000	-0.94078800

C	0.67600300	-1.33590100	0.03650300
C	-0.06459000	-1.82375300	1.11842400
C	-1.41134700	-1.55238000	1.13548100
H	0.41549300	-2.37970700	1.91473800
C	-1.93853800	-0.81333100	0.06904100
H	-2.04890600	-1.90317100	1.93779700
C	-3.38454600	-0.45790900	0.00944000
C	-4.07602900	-0.13752300	1.17841700
C	-5.42073900	0.19765800	1.08613300
H	-3.56666400	-0.12957300	2.13427700
C	-5.25097600	-0.13629000	-1.27063200
C	-6.02543700	0.19795700	-0.16329400
H	-5.98206900	0.46019500	1.97526600
H	-5.69041200	-0.14975700	-2.26322400
H	-7.07094900	0.45144900	-0.28607900
N	0.11316100	-0.64881500	-0.95584800
N	-1.17861500	-0.38959500	-0.94007200
N	2.87297900	-0.51661000	-0.50015300
N	-3.96058000	-0.45624500	-1.19641100
C	2.69162900	2.25743700	1.07636000
C	2.24996900	1.76686700	0.00616600
C	2.35939300	3.60574600	1.31160500
H	2.66862200	4.15559600	2.19438100
C	1.50468400	2.19703700	-1.05697400
C	1.58991600	4.22702500	0.32066300
H	1.19515800	1.62507700	-1.91962500
H	1.30565100	5.26705100	0.44408700
C	1.17245300	3.54415800	-0.83415300
H	0.57450500	4.06055000	-1.57746500

I1

C	4.79465600	-1.68696500	-0.32929700
C	3.96353800	-2.78063500	-0.13044500
C	2.60472600	-2.57247400	0.06507300
C	2.09580500	-1.28360900	0.06506700
C	4.24758000	-0.42035500	-0.29074200
H	5.85829600	-1.79851600	-0.48948600

H	4.36454400	-3.78671700	-0.13775600
H	1.92002100	-3.40184700	0.18776700
H	4.83960900	0.47989400	-0.38545800
C	0.62931900	-1.05488400	0.15378500
C	-0.12087100	-1.54208200	1.22513800
C	-1.47507800	-1.31621900	1.18288200
H	0.35741100	-2.05372200	2.05122100
C	-1.98654700	-0.63661300	0.06939000
H	-2.13167800	-1.65991500	1.97263500
C	-3.44209100	-0.34501500	-0.05801300
C	-4.17835400	0.04357200	1.06125200
C	-5.53095500	0.31786800	0.90418400
H	-3.69524000	0.15103900	2.02490800
C	-5.27823100	-0.20720300	-1.41074600
C	-6.09660800	0.18962700	-0.35691600
H	-6.12822800	0.63281700	1.75185700
H	-5.68713800	-0.32170500	-2.40980200
H	-7.14616100	0.39239900	-0.52943500
N	0.09002700	-0.43482500	-0.89012100
N	-1.21025500	-0.22576100	-0.93246400
N	2.93141800	-0.23155200	-0.09057700
N	-3.97965400	-0.46916200	-1.27492100
C	2.43972100	1.16112400	0.07607500
C	1.89291300	1.48394400	1.31275600
C	2.61013300	1.98696800	-1.03367400
H	3.03362100	1.61022000	-1.95992500
C	1.49741300	2.84181700	1.35299200
C	2.19122400	3.30634400	-0.92036700
H	1.05675300	3.23647400	2.26796100
H	2.29065100	3.98524000	-1.75939500
C	1.63275100	3.73185400	0.28566400
H	1.30052900	4.76106600	0.38713300

TS2

C	4.71144700	-1.98412200	0.09916600
C	3.72009800	-2.95977900	0.11389100
C	2.38724800	-2.57702500	0.07095600

C	2.05918300	-1.23239800	0.01874100
C	4.34315600	-0.65871200	0.02779000
H	5.76231500	-2.23859000	0.11489000
H	3.98395700	-4.00944800	0.15575600
H	1.58317400	-3.30073900	0.08487900
H	5.06176300	0.14510600	-0.04482900
C	0.63677000	-0.77621700	0.02732500
C	0.09935300	-0.20829600	1.22301500
C	-1.24863200	-0.04425300	1.26786500
H	0.75481500	0.10775400	2.02648300
C	-2.01324300	-0.52076300	0.17290000
H	-1.73587400	0.39458000	2.13032400
C	-3.47836700	-0.28327900	0.09532800
C	-4.01917500	0.89676000	0.61678400
C	-5.38815700	1.10670400	0.52824500
H	-3.37327500	1.64486900	1.06019100
C	-5.54849600	-1.00134600	-0.57254500
C	-6.17674400	0.13805900	-0.07926900
H	-5.82900900	2.01664000	0.91896300
H	-6.13096500	-1.78161600	-1.05350300
H	-7.24876700	0.25640700	-0.17496200
N	-0.16006800	-1.47583400	-0.80643100
N	-1.46375300	-1.27539600	-0.76805100
N	3.04385600	-0.30464300	-0.00473700
N	-4.23709700	-1.21729400	-0.49075600
C	2.61336700	1.08311700	-0.17691400
C	1.38967400	1.20015800	-0.81409800
C	3.38374900	2.12667900	0.33305800
H	4.31004800	1.96502400	0.87446200
C	0.93137600	2.51988900	-0.95069400
C	2.89665400	3.41612900	0.15176200
H	-0.02408200	2.71661500	-1.43248200
H	3.45943300	4.26189100	0.52890200
C	1.67469500	3.61082200	-0.49681500
H	1.30216600	4.62091900	-0.63819600

I2

C	-5.22464200	-0.78178000	0.49749000
C	-4.63054500	-2.05782300	0.23868600
C	-3.32124900	-2.15170200	-0.12219200
C	-2.53676400	-0.97091000	-0.25338200
C	-4.47841700	0.34358800	0.38009500
H	-6.26251300	-0.70159100	0.78970300
H	-5.23246000	-2.95297400	0.33802300
H	-2.85013000	-3.10890500	-0.30877500
H	-4.85712600	1.34054100	0.56137800
C	-1.20355800	-0.74918500	-0.58854300
C	-0.25928200	-1.79233300	-0.96218700
C	1.05706100	-1.83787900	-0.67535900
H	-0.66340500	-2.62372300	-1.53479000
C	1.77358700	-0.95995200	0.24454600
H	1.65578100	-2.63747900	-1.10101700
C	3.17966000	-0.57365900	0.15358900
C	3.92884000	-0.88360300	-0.99095300
C	5.26119500	-0.50495100	-1.03035400
H	3.47133400	-1.39561200	-1.82793800
C	4.98126300	0.45466600	1.13254100
C	5.80957000	0.17879100	0.05075000
H	5.86385300	-0.73392900	-1.90202400
H	5.36460700	0.99386300	1.99363900
H	6.84431800	0.49585600	0.05757500
N	0.54012700	-0.11602900	2.16691400
N	1.12200600	-0.50241200	1.28711200
N	-3.16141700	0.25326100	0.00942900
N	3.70175000	0.09050700	1.19616500
C	-2.23606400	1.27185700	-0.16988000
C	-1.01166900	0.67395700	-0.55122100
C	-2.40083700	2.65107400	-0.03783900
H	-3.34749200	3.08284200	0.26506900
C	0.07379100	1.51891300	-0.86093900
C	-1.30645600	3.45070500	-0.31477100
H	1.01518900	1.10453500	-1.20220500
H	-1.39332400	4.52651500	-0.22042000
C	-0.08435100	2.88545700	-0.73358800

H	0.74732700	3.54138100	-0.96449600

TS3a

C	-5.15050300	-1.14500600	0.83294900
C	-4.45711700	-2.35958900	0.52435000
C	-3.18514100	-2.32652300	0.04152400
C	-2.53886300	-1.07481800	-0.16942600
C	-4.53696400	0.04783900	0.63910600
H	-6.15883700	-1.16503900	1.22267200
H	-4.95376200	-3.30885400	0.68509500
H	-2.63842600	-3.23353400	-0.18568900
H	-4.99719800	1.00323200	0.85276300
C	-1.27497000	-0.72816300	-0.63377200
C	-0.27793000	-1.67838100	-1.11598600
C	1.05248400	-1.64688700	-0.90063300
H	-0.65372000	-2.50577200	-1.71265700
C	1.79474100	-0.77141700	-0.02468100
H	1.65886500	-2.39842400	-1.39695600
C	3.22792600	-0.56089300	0.02231100
C	4.17947800	-1.12858400	-0.85127700
C	5.50669200	-0.81331600	-0.66232000
H	3.86468500	-1.78745100	-1.65022700
C	4.90147500	0.57880100	1.18273500
C	5.88841000	0.05626900	0.37238600
H	6.25845100	-1.23575100	-1.31914500
H	5.12211000	1.25929600	1.99762000
H	6.92518700	0.31819400	0.53675700
N	1.77470900	0.65661700	1.74808300
N	1.26551100	-0.04085400	0.96432100
N	-3.25839700	0.08603300	0.14490100
N	3.61471700	0.26539700	0.99753400
C	-2.46299000	1.18774200	-0.13469400
C	-1.22734400	0.70615200	-0.62716400
C	-2.75351600	2.54785900	-0.01136000
H	-3.70639000	2.89222900	0.37320300
C	-0.26340400	1.64621700	-1.04357200
C	-1.77768400	3.44435900	-0.40775500

H	0.68445900	1.31362600	-1.45158200
H	-1.96509700	4.50840800	-0.32611900
C	-0.54605400	2.99286900	-0.92729700
H	0.19154000	3.72231600	-1.24214600

5a_cis

C	1.31053400	-0.77274200	-0.52194900
C	-1.78197900	-0.99578700	0.06285400
C	-3.14986100	-0.74244800	-0.09546900
C	-4.19287900	-1.12236100	-0.97478800
C	-4.68428000	0.64668500	1.15631400
C	-5.44133300	-0.61434300	-0.76361700
H	-3.97669500	-1.80063900	-1.79026300
C	-5.68814000	0.28413800	0.31922800
H	-4.76973100	1.31803800	1.99961500
H	-6.25859100	-0.88708000	-1.41944400
H	-6.67884800	0.68611400	0.48306200
C	2.62885100	-1.00832800	-0.14018600
C	3.38774200	-2.19772000	0.05285400
C	4.68644500	-2.11527200	0.45300500
H	2.90565900	-3.15259800	-0.11636600
C	4.57384000	0.29079100	0.51638600
C	5.29566300	-0.84278500	0.69369100
H	5.26765100	-3.01755700	0.60015000
H	4.96149400	1.28688700	0.68239800
H	6.32363000	-0.77196700	1.02108600
C	1.14773600	0.65353100	-0.54130500
C	0.09421500	1.51076300	-0.91509000
C	2.37257000	1.24031600	-0.14772400
C	0.28625000	2.87746700	-0.85732900
H	-0.85210500	1.10418800	-1.25086300
C	2.57337800	2.61970600	-0.08179300
C	1.51192800	3.43269700	-0.43535200
H	-0.52190900	3.54156800	-1.14226500
H	3.52294700	3.04063800	0.22742500
H	1.62657700	4.50937700	-0.39635700
N	3.26974000	0.21099500	0.10307900

N	-3.43615800	0.12478300	0.92947500
C	-0.94866800	-1.88830000	-0.73079200
H	-1.47771600	-2.72172500	-1.18384800
C	0.38392200	-1.82489700	-0.90976000
H	0.83532400	-2.66836200	-1.42869500
N	-2.34270500	0.38079000	1.66694900
N	-1.36277400	-0.29564600	1.14039600

TS3b (UM06-2X/6-311+G SMD=THF)**

C	1.99004200	3.50671100	0.15524700
C	0.63821600	3.14534400	-0.13633300
C	0.30947200	1.83541300	-0.36355100
C	1.30033900	0.83246300	-0.31223700
C	2.94797100	2.54785100	0.20931500
H	2.26418100	4.53697400	0.33696800
H	-0.12524400	3.91200100	-0.17527200
H	-0.70973800	1.54803200	-0.58527600
H	3.98805600	2.74944700	0.42712100
C	1.28376100	-0.58287800	-0.49220700
C	0.21708000	-1.42233800	-0.78161600
C	-1.16839200	-0.97807400	-0.98793600
H	0.42866600	-2.48773000	-0.85419400
C	-2.07919200	-0.85561900	0.05673000
H	-1.51840700	-0.70990000	-1.97961100
C	-3.50549500	-0.46295600	-0.04702600
C	-4.14620300	-0.40863600	-1.28771800
C	-5.47797000	-0.02278800	-1.32434700
H	-3.62374500	-0.67406700	-2.19705600
C	-5.40984600	0.18614600	1.04873500
C	-6.13005100	0.28487000	-0.13567300
H	-6.00174500	0.02935700	-2.27178800
H	-5.88086600	0.40849400	2.00106900
H	-7.16920200	0.58753500	-0.12069500
N	-1.31058600	-1.24984400	2.37344100
N	-1.66346100	-1.03729400	1.31291100
N	2.61665100	1.23706500	-0.01778600
N	-4.12834900	-0.17533800	1.10003600

C	3.44792900	0.12331100	-0.00352100
C	2.66305200	-1.00830600	-0.29018800
C	4.81930300	0.04348700	0.24061500
H	5.41158300	0.92337200	0.46153200
C	3.27438100	-2.26638400	-0.33343500
C	5.40021000	-1.21337200	0.19119900
H	2.69111400	-3.15401800	-0.55194000
H	6.46321100	-1.31410500	0.37618500
C	4.63547900	-2.35929700	-0.09331700
H	5.12184100	-3.32720400	-0.12367200

I3b

C	5.04164700	-2.04093000	-0.57620500
C	3.91943000	-2.92899100	-0.54765200
C	2.66292200	-2.45185400	-0.33293000
C	2.45744700	-1.05720200	-0.12880000
C	4.84992800	-0.71303500	-0.38853300
H	6.04184800	-2.41417500	-0.74714200
H	4.07799800	-3.98934200	-0.70243300
H	1.80637000	-3.11372900	-0.31731600
H	5.64610200	0.01902500	-0.39782100
C	1.32014200	-0.28318100	0.10417100
C	-0.01421600	-0.83764300	0.19408900
C	-1.17517200	-0.15885900	0.11205500
H	-0.05782700	-1.91656900	0.33578000
C	-2.49997700	-0.74540800	0.26986000
H	-1.17659900	0.90621100	-0.07940100
C	-3.78248900	-0.12302700	-0.06616100
C	-3.84082700	1.10908900	-0.73303400
C	-5.08496900	1.65136100	-1.01228500
H	-2.93844300	1.62202900	-1.03783300
C	-6.07316300	-0.26017700	0.00683700
C	-6.23338900	0.96103800	-0.63666800
H	-5.15634800	2.60300800	-1.52660200
H	-6.94015300	-0.83866900	0.31147700
H	-7.22301300	1.35002500	-0.83826800
N	-2.62534800	-2.99067400	1.20494500

N	-2.58072300	-1.95264500	0.77231300
N	3.58494900	-0.23022100	-0.16958500
N	-4.88738900	-0.79506700	0.29177800
C	3.18314200	1.07985700	0.04431300
C	1.77797600	1.07574200	0.22194600
C	3.96055800	2.23661800	0.10731300
H	5.03422000	2.20584300	-0.03656500
C	1.15005200	2.30688400	0.50182600
C	3.30840700	3.42841600	0.36616700
H	0.08433900	2.36383800	0.68058800
H	3.87684800	4.34902900	0.42158400
C	1.91452800	3.45648600	0.56886200
H	1.43238300	4.40262500	0.78677100

TS4b

C	-5.10207300	-2.15111800	0.29250900
C	-3.96355900	-3.01796000	0.23740600
C	-2.70868800	-2.50617800	0.11319500
C	-2.51997200	-1.09636500	0.02968200
C	-4.92747900	-0.80983200	0.21962200
H	-6.10129000	-2.55129600	0.39460100
H	-4.10927100	-4.08954300	0.29944600
H	-1.83992600	-3.15138200	0.07940700
H	-5.73758800	-0.09417600	0.25898600
C	-1.38790300	-0.29120300	-0.09550100
C	-0.04103600	-0.81733100	-0.17663800
C	1.10791600	-0.13291700	-0.01237200
H	0.03346800	-1.88351400	-0.38384500
C	2.43224000	-0.70208400	-0.11790000
H	1.08814700	0.92004100	0.24015100
C	3.71765200	-0.05128400	0.05397500
C	3.91615500	1.31238200	0.35660300
C	5.20882400	1.77092200	0.48361500
H	3.07089700	1.97636100	0.48296000
C	6.01821300	-0.42706300	0.01680600
C	6.29084600	0.89315600	0.31306700
H	5.38882800	2.81439700	0.71541200

H	6.80484600	-1.15920100	-0.12872200
H	7.31404400	1.23157500	0.40832700
N	3.67891700	-2.57364700	-0.51529000
N	2.68126000	-1.98132900	-0.40160700
N	-3.66394700	-0.29120700	0.08938300
N	4.76238900	-0.86889700	-0.10518300
C	-3.27663200	1.03663900	-0.00505400
C	-1.86529400	1.06562200	-0.12680600
C	-4.06920100	2.18513200	-0.00912100
H	-5.14716600	2.12993800	0.08697000
C	-1.24789600	2.32346000	-0.28643900
C	-3.42735600	3.40222500	-0.14835500
H	-0.17767400	2.40858000	-0.42057300
H	-4.00866300	4.31645700	-0.15489300
C	-2.02744300	3.46478600	-0.29343100
H	-1.55287600	4.43138900	-0.41851200

5a

C	-1.23444100	0.03330700	-0.07478900
C	2.47734400	-0.88430600	-0.50672200
C	3.37983700	0.01863500	0.07767000
C	3.34992800	1.15160600	0.92852900
C	5.79973200	0.13486300	0.00210800
C	4.52910300	1.74215800	1.28012000
H	2.40151300	1.52072000	1.29398000
C	5.77185500	1.23062500	0.80046000
H	6.68511300	-0.33619600	-0.40186600
H	4.52942200	2.60581400	1.93319100
H	6.70439900	1.70537900	1.07415300
C	-2.08617900	-1.05184900	0.13886600
C	-1.87864100	-2.43716500	0.39799400
C	-2.94459300	-3.26178100	0.59592500
H	-0.86686700	-2.81480200	0.44961200
C	-4.48421900	-1.43320800	0.32411700
C	-4.27986600	-2.75485900	0.54389300
H	-2.78332800	-4.31336400	0.79996700
H	-5.46255100	-0.97317900	0.28733700

H	-5.12967800	-3.40753000	0.68783300
C	-2.07795600	1.18920700	-0.18889000
C	-1.81234600	2.55446600	-0.40625000
C	-3.42160900	0.77593500	-0.06239400
C	-2.87187500	3.43781900	-0.47842000
H	-0.79247700	2.90500600	-0.51993400
C	-4.49813000	1.66152500	-0.13949500
C	-4.20586800	2.99762900	-0.34687500
H	-2.67937900	4.49166300	-0.64423300
H	-5.52239100	1.32204300	-0.04068300
H	-5.01370300	3.71668300	-0.41174700
N	-3.41420600	-0.59757400	0.13699000
N	4.60572900	-0.45070700	-0.33406100
C	0.21136400	0.06372900	-0.15484600
H	0.65444700	1.03714200	0.04508600
C	1.02703900	-0.95614800	-0.48030500
H	0.62305400	-1.91337700	-0.79333500
N	4.48089400	-1.55194400	-1.08896900
N	3.20609000	-1.80250700	-1.18018100

TS (5a → 5a_cis) (UM06-2X/6-311+G SMD=THF)**

C	1.05456200	-0.22317900	0.96435200
C	-2.35559100	0.08618600	1.35487700
C	-2.64675300	-0.51075500	0.10192900
C	-1.99707500	-1.31835100	-0.85477300
C	-4.64159800	-0.46463200	-1.26722400
C	-2.68050300	-1.67919800	-1.98459600
H	-0.97660000	-1.63080800	-0.67721100
C	-4.02232600	-1.24583200	-2.18913200
H	-5.65133900	-0.08360500	-1.33135900
H	-2.20268800	-2.29721700	-2.73411200
H	-4.56292100	-1.53126200	-3.08158200
C	2.21228500	-0.95443200	0.57368500
C	2.64623800	-2.26912600	0.83454100
C	3.83094000	-2.71724700	0.31602100
H	2.02596200	-2.90747900	1.45222900
C	4.21556900	-0.58311300	-0.74605700

C	4.63104800	-1.84962000	-0.49252300
H	4.16868800	-3.72637400	0.51527200
H	4.77233500	0.12309800	-1.34702600
H	5.57056300	-2.18812800	-0.90764600
C	1.20984600	1.09106500	0.35353600
C	0.42158700	2.25209400	0.35799600
C	2.42483500	1.10558900	-0.36542800
C	0.85743600	3.36610600	-0.34031400
H	-0.51497100	2.28226300	0.90114800
C	2.87266400	2.22223300	-1.07168400
C	2.07200100	3.35174400	-1.04923600
H	0.25351800	4.26591600	-0.34089000
H	3.80820300	2.21159600	-1.61773200
H	2.38766300	4.23822200	-1.58670200
N	3.02669100	-0.13844100	-0.22638500
N	-3.93902200	-0.11340100	-0.14411200
C	-1.17391600	0.06226000	2.12173900
H	-1.18900800	0.63637300	3.04604400
C	0.01058100	-0.71421700	1.74024100
H	0.08024500	-1.74292200	2.09240700
N	-4.41609900	0.66012200	0.84736000
N	-3.47451500	0.77813300	1.73306200

Figure S3a

TS1

C	4.82096400	-1.93477000	0.34512000
C	4.19166100	-2.39547000	-0.80287800
C	2.81675300	-2.23593300	-0.92140600
C	2.12803300	-1.61028800	0.11695000
C	4.04244500	-1.33296000	1.33034400
H	5.89033700	-2.03477800	0.48262400
H	4.75964100	-2.86473800	-1.59761000
H	2.29158500	-2.56342100	-1.81055100
H	4.50054700	-0.96781900	2.24422600
C	0.66106300	-1.38731100	0.01638500
C	-0.20159700	-2.33975700	-0.53750400

C	-1.54237200	-2.04242700	-0.56849100
H	0.18202400	-3.27927200	-0.91599700
C	-1.94814100	-0.80497100	-0.05070200
H	-2.26344600	-2.73123400	-0.99162000
C	-3.38108700	-0.39756100	-0.03043800
C	-4.37649700	-1.34280600	0.21881900
C	-5.70110800	-0.92504100	0.23817400
H	-4.11810300	-2.37635500	0.41477500
C	-4.91979000	1.28024800	-0.23300200
C	-5.98318300	0.41383900	0.00564300
H	-6.49622900	-1.63390900	0.43741800
H	-5.10251100	2.33352500	-0.42185100
H	-6.99959100	0.78694400	0.00905200
N	0.20661500	-0.22421700	0.47756300
N	-1.07442100	0.06931900	0.44387700
N	2.72559800	-1.16844700	1.22734600
N	-3.64587500	0.89365000	-0.25040400
C	2.49272500	1.79395800	-0.72917600
C	1.49199600	1.79033900	0.02904600
C	3.04697400	3.05672300	-1.00899000
H	3.90951500	3.20948500	-1.64899300
C	0.74215900	2.71572600	0.69756000
C	2.40954100	4.14562300	-0.39961100
H	-0.12583300	2.54136700	1.31824800
H	2.79048600	5.14679800	-0.57322200
C	1.28845200	3.98391400	0.43135300
H	0.82421400	4.85481800	0.88166300

I1

C	4.88997700	-1.62862500	0.44134900
C	4.30549800	-2.23787100	-0.66088000
C	2.94277200	-2.07441900	-0.87188600
C	2.23639900	-1.28403300	0.02834500
C	4.08116800	-0.89055600	1.30209100
H	5.94975200	-1.72344900	0.64165500
H	4.89980800	-2.82448800	-1.35124800
H	2.44413300	-2.51236400	-1.72808900

H	4.50092800	-0.42053600	2.18541100
C	0.76860900	-1.10177000	-0.13154000
C	-0.08023300	-2.20526800	-0.26828900
C	-1.43522400	-2.00590300	-0.26711500
H	0.35210500	-3.19448400	-0.34841400
C	-1.90754800	-0.69173400	-0.10332800
H	-2.12517600	-2.83110200	-0.38866600
C	-3.36137300	-0.37352100	-0.03528700
C	-4.25387300	-1.29032800	0.51670600
C	-5.60070200	-0.95069300	0.56942400
H	-3.90615000	-2.23584400	0.91453200
C	-5.02778600	1.12602400	-0.46153600
C	-5.99936200	0.27992400	0.06927800
H	-6.32106100	-1.63659700	0.99914300
H	-5.30390200	2.09520600	-0.86438600
H	-7.03725600	0.58755100	0.08660800
N	0.21608000	0.11788900	-0.06731000
N	-1.08195100	0.33535700	-0.02539700
N	2.77590700	-0.71731900	1.10788200
N	-3.73556100	0.81531900	-0.51428100
C	1.92626200	1.44395000	-1.19462600
C	1.04001600	1.34769000	-0.12909300
C	2.62381400	2.67457200	-1.16063900
H	3.34633300	2.89401300	-1.94601300
C	0.79252300	2.27630100	0.88064600
C	2.44911700	3.64828100	-0.17613500
H	0.06221400	2.08443300	1.65980000
H	3.02725800	4.56747800	-0.20845600
C	1.52751000	3.45267300	0.85546500
H	1.38429600	4.20330000	1.62416300

TS2

C	-4.87465300	-1.61911800	0.58519100
C	-4.34516000	-2.24875000	-0.55931100
C	-3.00655900	-2.10107400	-0.82826400
C	-2.21913400	-1.32912600	0.05561100
C	-4.05302200	-0.84070900	1.36348700

H	-5.91589600	-1.74377700	0.85673100
H	-4.98102700	-2.83320300	-1.21303000
H	-2.55954500	-2.54658500	-1.70918500
H	-4.43746600	-0.31426500	2.23068500
C	-0.81382900	-1.12838500	-0.16521000
C	0.04805000	-2.12135400	-0.66001000
C	1.39496100	-1.91214000	-0.63519300
H	-0.37676900	-3.06524500	-0.97858900
C	1.85950300	-0.68232800	-0.09564200
H	2.09207100	-2.67848200	-0.94617200
C	3.31504500	-0.41384800	0.08523200
C	4.24175200	-0.91583500	-0.82847500
C	5.58704800	-0.62855600	-0.63291700
H	3.92114600	-1.49717400	-1.68428800
C	4.95499700	0.59145700	1.31843300
C	5.95584400	0.13853200	0.46296500
H	6.33126100	-0.99586600	-1.32974500
H	5.20536100	1.19039000	2.18839000
H	6.99194700	0.38481500	0.65829400
N	-0.27550000	0.08935200	0.15819200
N	1.04983400	0.28159800	0.25806200
N	-2.73266200	-0.66048000	1.11557400
N	3.66254600	0.32749700	1.14042900
C	-2.32285400	1.52646400	0.44506600
C	-1.03342800	1.33899000	-0.00666700
C	-2.97044500	2.71226000	0.10086200
H	-4.00386000	2.87349500	0.40299700
C	-0.35620300	2.32881000	-0.73843200
C	-2.30212200	3.73876200	-0.56693600
H	0.63726500	2.14227200	-1.13154100
H	-2.79503700	4.68808600	-0.75343900
C	-0.98891000	3.53647500	-0.99172400
H	-0.46426700	4.30661300	-1.54530800

P2

C	-0.77251500	-1.12843000	-0.29762000
C	0.17181800	-2.10970800	-0.76481600

C	1.49077600	-1.87444600	-0.67569100
C	1.94252100	-0.58689600	-0.14868500
H	-0.19911100	-3.04123800	-1.17357400
H	2.21471000	-2.59519600	-1.03084900
C	3.39285300	-0.30516100	0.02617100
C	4.28157300	-1.34980400	0.29231100
C	5.08580600	1.23468900	0.09856000
C	5.62809400	-1.05708000	0.46809300
H	3.92413800	-2.36780800	0.38353900
C	6.04585000	0.26241900	0.36817900
H	5.37447100	2.27773300	0.00932600
H	6.33580200	-1.84910900	0.68389900
H	7.08478900	0.54035900	0.49326500
C	-2.10970100	-1.30465500	-0.15164700
C	-2.84929200	-2.51655300	-0.44645300
C	-4.11776600	-2.66846100	-0.01535800
H	-2.35529200	-3.30401600	-1.00117300
C	-4.14307600	-0.42688400	0.85437300
C	-4.76816900	-1.61498400	0.73542600
H	-4.65668600	-3.58423200	-0.22769900
H	-4.58460100	0.42038500	1.36312600
H	-5.74187100	-1.75974800	1.18120800
C	-1.02062500	1.28494900	-0.03175700
C	-0.50352800	2.55558500	-0.24526800
C	-2.40804000	1.10072700	0.08113800
C	-1.36588800	3.65204300	-0.32288900
H	0.56724400	2.68033700	-0.33357200
C	-3.26007500	2.18534600	-0.06758500
C	-2.73619400	3.46978600	-0.24354100
H	-0.95201400	4.64344900	-0.46331400
H	-4.33229100	2.03788300	-0.03981200
H	-3.41068600	4.31329400	-0.32660900
N	-0.20485600	0.13726000	0.06151600
N	-2.87982400	-0.21328500	0.34259200
N	1.12333800	0.36056200	0.15290000
N	3.79346500	0.96875300	-0.07063700

Figure S3b

TS3

C	4.84644100	-1.85437600	-0.30088000
C	4.08978900	-2.92599100	0.15553100
C	2.71498700	-2.77314400	0.28175200
C	2.14456400	-1.54948900	-0.06586600
C	4.19125100	-0.66684100	-0.60758700
H	5.92069400	-1.92746800	-0.41345900
H	4.56116700	-3.86863800	0.40776300
H	2.09023300	-3.58990300	0.62294300
H	4.74560400	0.20542000	-0.94078800
C	0.67600300	-1.33590100	0.03650300
C	-0.06459000	-1.82375300	1.11842400
C	-1.41134700	-1.55238000	1.13548100
H	0.41549300	-2.37970700	1.91473800
C	-1.93853800	-0.81333100	0.06904100
H	-2.04890600	-1.90317100	1.93779700
C	-3.38454600	-0.45790900	0.00944000
C	-4.07602900	-0.13752300	1.17841700
C	-5.42073900	0.19765800	1.08613300
H	-3.56666400	-0.12957300	2.13427700
C	-5.25097600	-0.13629000	-1.27063200
C	-6.02543700	0.19795700	-0.16329400
H	-5.98206900	0.46019500	1.97526600
H	-5.69041200	-0.14975700	-2.26322400
H	-7.07094900	0.45144900	-0.28607900
N	0.11316100	-0.64881500	-0.95584800
N	-1.17861500	-0.38959500	-0.94007200
N	2.87297900	-0.51661000	-0.50015300
N	-3.96058000	-0.45624500	-1.19641100
C	2.69162900	2.25743700	1.07636000
C	2.24996900	1.76686700	0.00616600
C	2.35939300	3.60574600	1.31160500
H	2.66862200	4.15559600	2.19438100
C	1.50468400	2.19703700	-1.05697400
C	1.58991600	4.22702500	0.32066300

H	1.19515800	1.62507700	-1.91962500
H	1.30565100	5.26705100	0.44408700
C	1.17245300	3.54415800	-0.83415300
H	0.57450500	4.06055000	-1.57746500

I3

C	4.79465600	-1.68696500	-0.32929700
C	3.96353800	-2.78063500	-0.13044500
C	2.60472600	-2.57247400	0.06507300
C	2.09580500	-1.28360900	0.06506700
C	4.24758000	-0.42035500	-0.29074200
H	5.85829600	-1.79851600	-0.48948600
H	4.36454400	-3.78671700	-0.13775600
H	1.92002100	-3.40184700	0.18776700
H	4.83960900	0.47989400	-0.38545800
C	0.62931900	-1.05488400	0.15378500
C	-0.12087100	-1.54208200	1.22513800
C	-1.47507800	-1.31621900	1.18288200
H	0.35741100	-2.05372200	2.05122100
C	-1.98654700	-0.63661300	0.06939000
H	-2.13167800	-1.65991500	1.97263500
C	-3.44209100	-0.34501500	-0.05801300
C	-4.17835400	0.04357200	1.06125200
C	-5.53095500	0.31786800	0.90418400
H	-3.69524000	0.15103900	2.02490800
C	-5.27823100	-0.20720300	-1.41074600
C	-6.09660800	0.18962700	-0.35691600
H	-6.12822800	0.63281700	1.75185700
H	-5.68713800	-0.32170500	-2.40980200
H	-7.14616100	0.39239900	-0.52943500
N	0.09002700	-0.43482500	-0.89012100
N	-1.21025500	-0.22576100	-0.93246400
N	2.93141800	-0.23155200	-0.09057700
N	-3.97965400	-0.46916200	-1.27492100
C	2.43972100	1.16112400	0.07607500
C	1.89291300	1.48394400	1.31275600
C	2.61013300	1.98696800	-1.03367400

H	3.03362100	1.61022000	-1.95992500
C	1.49741300	2.84181700	1.35299200
C	2.19122400	3.30634400	-0.92036700
H	1.05675300	3.23647400	2.26796100
H	2.29065100	3.98524000	-1.75939500
C	1.63275100	3.73185400	0.28566400
H	1.30052900	4.76106600	0.38713300

TS4

C	0.67143100	-1.16318700	0.02056000
C	-0.04913400	-1.57925600	1.13195800
C	-1.39369300	-1.26749800	1.17997800
C	-1.93623300	-0.63568500	0.04803900
H	0.44675200	-2.10010600	1.94343100
H	-2.00746800	-1.50656600	2.03799500
C	-3.39817600	-0.33967600	-0.02454400
C	-4.32571000	-1.23859400	0.50547100
C	-5.05809800	1.08911500	-0.68422200
C	-5.67777100	-0.93323100	0.41930500
H	-3.99392400	-2.16580300	0.95657200
C	-6.05669600	0.25700400	-0.18647800
H	-5.31628300	2.02948700	-1.16163300
H	-6.42052200	-1.61676900	0.81416500
H	-7.09792200	0.54033200	-0.27678000
C	2.11703100	-1.31441900	-0.05235800
C	2.76120200	-2.53158600	0.16975000
C	4.11792400	-2.65232300	-0.04988800
H	2.15611700	-3.38249700	0.45774600
C	4.18861500	-0.34155400	-0.64363900
C	4.83591500	-1.54204500	-0.50982200
H	4.61229000	-3.60555700	0.09126800
H	4.68889300	0.56004200	-0.96883300
H	5.88570300	-1.60760900	-0.76094300
C	1.06111000	1.49953200	-0.53845600
C	0.62157800	2.77133700	-0.16478900
C	2.33172400	1.13996900	-0.12935100
C	1.44519900	3.66470800	0.52146600

H	-0.38431100	3.09010900	-0.43495400
C	3.16986500	1.98640300	0.60835700
C	2.72329100	3.26548100	0.91348800
H	1.09471600	4.66398400	0.76180900
H	4.13488900	1.64787500	0.97453500
H	3.36071100	3.93418800	1.48015100
N	0.11089500	-0.46519100	-1.01672800
N	2.86402200	-0.21322900	-0.36852400
N	-1.22412000	-0.31148900	-1.02062900
N	-3.75877900	0.80589600	-0.61175700

P4

C	-0.77251500	-1.12843000	-0.29762000
C	0.17181800	-2.10970800	-0.76481600
C	1.49077600	-1.87444600	-0.67569100
C	1.94252100	-0.58689600	-0.14868500
H	-0.19911100	-3.04123800	-1.17357400
H	2.21471000	-2.59519600	-1.03084900
C	3.39285300	-0.30516100	0.02617100
C	4.28157300	-1.34980400	0.29231100
C	5.08580600	1.23468900	0.09856000
C	5.62809400	-1.05708000	0.46809300
H	3.92413800	-2.36780800	0.38353900
C	6.04585000	0.26241900	0.36817900
H	5.37447100	2.27773300	0.00932600
H	6.33580200	-1.84910900	0.68389900
H	7.08478900	0.54035900	0.49326500
C	-2.10970100	-1.30465500	-0.15164700
C	-2.84929200	-2.51655300	-0.44645300
C	-4.11776600	-2.66846100	-0.01535800
H	-2.35529200	-3.30401600	-1.00117300
C	-4.14307600	-0.42688400	0.85437300
C	-4.76816900	-1.61498400	0.73542600
H	-4.65668600	-3.58423200	-0.22769900
H	-4.58460100	0.42038500	1.36312600
H	-5.74187100	-1.75974800	1.18120800
C	-1.02062500	1.28494900	-0.03175700

C	-0.50352800	2.55558500	-0.24526800
C	-2.40804000	1.10072700	0.08113800
C	-1.36588800	3.65204300	-0.32288900
H	0.56724400	2.68033700	-0.33357200
C	-3.26007500	2.18534600	-0.06758500
C	-2.73619400	3.46978600	-0.24354100
H	-0.95201400	4.64344900	-0.46331400
H	-4.33229100	2.03788300	-0.03981200
H	-3.41068600	4.31329400	-0.32660900
N	-0.20485600	0.13726000	0.06151600
N	-2.87982400	-0.21328500	0.34259200
N	1.12333800	0.36056200	0.15290000
N	3.79346500	0.96875300	-0.07063700

Figure S3c

TS5

C	4.14881000	-1.31498400	-1.39474500
C	4.64579000	-2.13557300	-0.38959300
C	3.77207700	-2.57551300	0.59940900
N	2.47916900	-2.24992500	0.63408900
C	2.01157500	-1.45368500	-0.33223900
C	2.80676200	-0.96157800	-1.36734800
H	4.79526800	-0.95206700	-2.18524600
H	5.68660600	-2.43328500	-0.36638300
H	4.12601600	-3.21544600	1.40153500
H	2.37450700	-0.31421400	-2.11985000
C	-1.87505500	0.05691800	-0.10650000
C	0.58538300	-1.02616200	-0.23552100
C	-4.43417600	2.74156900	0.50938400
C	-3.23290700	2.04718000	0.54074400
C	-3.16687100	0.80425500	-0.08915100
N	-4.20554600	0.25254400	-0.72376800
C	-5.35051000	0.93518100	-0.75500600
C	-5.51714000	2.17805200	-0.15375900
H	-4.52174100	3.70726900	0.99342300
H	-2.35856700	2.44813100	1.03686900

H	-6.17242500	0.46644700	-1.28684600
H	-6.47231300	2.68506500	-0.20858900
N	-0.82745000	0.80165400	-0.35841600
N	0.39873800	0.25733700	-0.40985500
C	-0.49402500	-1.92781200	0.03661400
C	-0.33220000	-3.32356200	0.20003000
C	-1.78265700	-1.35509400	0.13243200
C	-1.42187700	-4.10330900	0.49669200
C	-2.88483400	-2.17661500	0.46789400
C	-2.69879000	-3.52417900	0.65157500
H	0.64878400	-3.76628200	0.10058200
H	-1.30097700	-5.17285800	0.62265500
H	-3.86860000	-1.74166500	0.56914500
H	-3.54284200	-4.15307300	0.90951100
C	1.86863500	3.26530300	-0.64258500
C	2.03559900	2.16514300	-0.07143500
C	2.74638400	4.30911500	-0.31122900
H	2.70360900	5.30477500	-0.73873800
C	2.90205200	1.66889600	0.86491100
C	3.71719200	3.97760900	0.64407000
H	2.93288300	0.67434400	1.29062000
H	4.43204300	4.73245000	0.95506000
C	3.79454300	2.69734900	1.21787700
H	4.56080600	2.48811600	1.95677700

I5

C	-4.43068900	0.80189400	-1.07029500
C	-4.93914900	1.45804600	0.04367600
C	-4.06408800	1.82195500	1.06198800
N	-2.75467900	1.56976900	1.02341300
C	-2.28915300	0.91973200	-0.04276500
C	-3.07208900	0.51820300	-1.11957800
H	-5.07955700	0.50732400	-1.88637500
H	-5.99355100	1.68964400	0.12726000
H	-4.42624300	2.33978800	1.94387900
H	-2.62831600	-0.01255400	-1.95297200
C	1.82219900	-0.00273200	-0.01708100

C	-0.81786200	0.65969800	-0.03206500
C	4.84557600	-2.15870500	-0.58479900
C	3.54788800	-1.67465600	-0.68345300
C	3.21903500	-0.52534200	0.03291500
N	4.08336100	0.13147300	0.81047300
C	5.32193200	-0.35113400	0.90405300
C	5.75163700	-1.48780600	0.22605800
H	5.14083300	-3.04672400	-1.13107600
H	2.80164100	-2.16606400	-1.29492900
H	6.00105800	0.19445200	1.55120400
H	6.77220700	-1.83220100	0.33679000
N	0.91244900	-0.93531800	0.01769300
N	-0.38384100	-0.58457400	0.00339300
C	0.12553200	1.72680500	-0.09308500
C	-0.28101900	3.07516100	-0.20410200
C	1.49978300	1.39502900	-0.10321200
C	0.67129000	4.05671000	-0.32359200
C	2.45699600	2.42029100	-0.24488200
C	2.04056900	3.72662500	-0.35046100
H	-1.33327500	3.32589000	-0.20150500
H	0.36868500	5.09318400	-0.41098000
H	3.50861600	2.17607500	-0.27183400
H	2.77574200	4.51457900	-0.46332600
C	-1.31489600	-2.55961900	-1.09747100
C	-1.30579100	-1.74912900	0.02972700
C	-2.25443400	-3.60860800	-0.95405300
H	-2.37697700	-4.32310000	-1.76751000
C	-2.06059100	-1.87437500	1.19546800
C	-3.05575600	-3.79892400	0.17377600
H	-1.95393400	-1.17344100	2.01834100
H	-3.75737700	-4.62750200	0.21127800
C	-2.95907000	-2.93125800	1.26302800
H	-3.56768400	-3.07596100	2.14817300

TS6

C	-4.40219700	1.81044800	-0.55925700
C	-5.01671800	1.19290900	0.55062100

C	-4.25313500	0.43368700	1.39949500
N	-2.91188200	0.25801700	1.25932600
C	-2.31294800	0.95651500	0.26046600
C	-3.04154400	1.69492300	-0.70140000
H	-4.99032400	2.35859700	-1.28506400
H	-6.07883600	1.30424300	0.73270800
H	-4.70210400	-0.09982000	2.23071500
H	-2.52953900	2.13045500	-1.55135700
C	1.76305100	0.05410800	0.12407500
C	-0.89127000	0.74520800	0.13343300
C	4.81787200	-1.80825100	1.28495400
C	3.52036900	-1.31480400	1.25414400
C	3.16405600	-0.45668300	0.21513700
N	4.00803900	-0.08617800	-0.75145200
C	5.24788200	-0.57484100	-0.71451500
C	5.70132000	-1.43478100	0.28046700
H	5.13180500	-2.47338900	2.08082800
H	2.79457700	-1.57999200	2.01213200
H	5.90856900	-0.26482200	-1.51779100
H	6.72152900	-1.79692600	0.26226800
N	0.86446200	-0.85731500	0.31811000
N	-0.45098600	-0.52838400	0.26756500
C	0.07051000	1.78540900	-0.09899600
C	-0.29877000	3.14481200	-0.20024500
C	1.44077000	1.44162800	-0.12885100
C	0.66584600	4.10863200	-0.38712600
C	2.41168500	2.44480700	-0.29772000
C	2.02471600	3.75973000	-0.44326200
H	-1.33531100	3.43622300	-0.10324500
H	0.37410300	5.14932900	-0.46453800
H	3.45907800	2.18051100	-0.31191900
H	2.77439300	4.53077300	-0.57426200
C	-2.57117700	-1.85583800	0.47228600
C	-1.29894900	-1.68657400	-0.03868600
C	-3.32683700	-2.92492700	-0.01085200
H	-4.35067800	-3.06208300	0.33405200
C	-0.74001500	-2.59104000	-0.95677400

C	-2.78508200	-3.86445700	-0.88683000
H	0.24638300	-2.42212600	-1.37385800
H	-3.36581000	-4.72733900	-1.19847400
C	-1.48423300	-3.68968600	-1.36064500
H	-1.05647500	-4.39220900	-2.06654800

P6

C	-3.67822700	2.53725500	1.42702200
C	-4.46337500	1.39030300	1.81118900
C	-4.07678800	0.17343900	1.38171800
N	-2.95538100	-0.00899400	0.59756400
C	-2.05448200	1.06600300	0.35395700
C	-2.53679400	2.38308100	0.72548000
H	-3.99014100	3.52787100	1.73615700
H	-5.33341100	1.48662500	2.44462700
H	-4.61234200	-0.72896800	1.64134700
H	-1.91682600	3.23710500	0.49616300
N	0.78936700	-0.99507800	-0.03210800
C	1.74199700	-0.11814500	0.00123000
C	-0.83487600	0.78725600	-0.16953600
N	-0.45395500	-0.58142500	-0.29577200
C	4.67876500	-2.42752700	0.47874800
C	3.44530100	-1.95622700	0.05900300
C	3.07481100	-0.64844300	0.39152400
N	3.87281900	0.16605500	1.09129000
C	5.05713600	-0.30141500	1.49003300
C	5.50790200	-1.58600000	1.21402300
H	4.99228400	-3.43538300	0.23151400
H	2.77148200	-2.57663600	-0.51751600
H	5.67404300	0.38664600	2.06003600
H	6.48007900	-1.91102800	1.56270000
C	-2.74952200	-1.24094200	-0.07908400
C	-3.77764900	-2.14479600	-0.32876100
C	-1.45176300	-1.54682700	-0.51059200
C	-3.49894400	-3.37095200	-0.93288900
C	-1.18142800	-2.75534200	-1.13789600
C	-2.20480100	-3.68221700	-1.32533100

H	-4.79642500	-1.90662400	-0.05264100
H	-4.30644800	-4.07296500	-1.10172600
H	-0.16903600	-2.96124700	-1.46253900
H	-1.98805300	-4.63348600	-1.79608200
C	0.20949700	1.70834400	-0.62871000
C	1.53858800	1.26891400	-0.46137100
C	-0.03737700	2.93468600	-1.24893800
C	2.59032000	2.08921700	-0.87749600
C	1.01921200	3.75107800	-1.63786500
C	2.33201800	3.33198500	-1.44532800
H	-1.05598800	3.24287600	-1.44995300
H	3.61206100	1.75467900	-0.75945000
H	0.81473900	4.70707500	-2.10601800
H	3.15692600	3.96319300	-1.75420900

Figure S3d

TS7

C	5.52374300	0.04450300	0.85447200
C	4.74926200	-0.43384300	1.90653400
N	3.43318400	-0.62670100	1.82370400
C	2.83256300	-0.34658500	0.66437900
C	3.52346900	0.13236900	-0.44996600
H	5.45638300	0.70964400	-1.19509000
H	6.58979700	0.18466000	0.98261700
H	5.20832300	-0.67567000	2.86006300
H	2.99019000	0.35356400	-1.36729500
C	-1.30532400	-0.57479400	0.59269400
C	1.35316600	-0.55231000	0.60301700
C	-4.80812000	-0.32540800	1.95717700
C	-3.43317500	-0.51100500	1.91344800
C	-2.78620300	-0.40357000	0.68147600
N	-3.43274400	-0.12874500	-0.45622700
C	-4.75225200	0.05029900	-0.39866200
C	-5.48607100	-0.03650800	0.77966500
H	-5.34075300	-0.40679600	2.89755900
H	-2.86392300	-0.73622000	2.80521500

H	-5.24570400	0.27726600	-1.33872400
H	-6.55755700	0.11920700	0.76698700
N	-0.63505100	-0.24158800	1.70039100
N	0.67629800	-0.22059100	1.70432200
C	0.73769200	-1.32818400	-0.44055800
C	1.45261400	-2.08900100	-1.39058400
C	-0.67082200	-1.33863300	-0.45157900
C	0.76662700	-2.83099900	-2.32307500
C	-1.35646500	-2.10654800	-1.41636900
C	-0.64153100	-2.83877400	-2.33542700
H	2.53479200	-2.10804600	-1.36873800
H	1.31234400	-3.42537200	-3.04647400
H	-2.43709000	-2.12449000	-1.42316000
H	-1.16644600	-3.43727300	-3.07078400
C	0.58948500	1.65625400	-0.35277400
C	-0.66666900	1.58393200	-0.37675700
C	1.34502400	2.74240300	-0.75876500
H	2.42607900	2.81380100	-0.73861700
C	-1.54997100	2.55169600	-0.81384600
C	0.53333100	3.79512400	-1.21492300
H	-2.62920400	2.46984400	-0.83759900
H	1.00711000	4.70996200	-1.55533000
C	-0.86615500	3.70253700	-1.24235100
H	-1.44269100	4.54748100	-1.60412200

P7

C	5.51227100	-0.07571000	0.56481000
C	4.79501000	-0.02560200	-0.62628900
C	2.78413600	-0.02296000	0.47350800
C	3.41064700	-0.07260000	1.71605400
C	4.80100100	-0.09957000	1.75550200
H	6.59484500	-0.09551800	0.55000200
H	5.31238100	-0.00561300	-1.58049800
H	2.82574200	-0.09054300	2.62453500
H	5.31713800	-0.13937000	2.70781000
C	1.28038100	-0.00039800	0.32159100
C	0.68709200	-1.20844400	-0.37971400

C	1.38107500	-2.28511100	-0.91683000
C	-0.70791300	-1.19607900	-0.37054300
C	0.66219900	-3.34332300	-1.46937100
H	2.46332500	-2.30384500	-0.91409700
C	-1.42406100	-2.26537200	-0.89469800
C	-1.28018800	0.02578200	0.33297200
C	-0.72910500	-3.33407500	-1.45770700
H	1.19412500	-4.18270200	-1.90190300
H	-2.50676800	-2.27900300	-0.86701200
H	-1.27998200	-4.16700800	-1.87879400
C	-2.78899400	0.01273000	0.46428800
C	-3.56743000	0.10510700	-0.69319800
C	-4.65270800	-0.15684900	1.77838300
C	-4.94837500	0.06754100	-0.57605200
H	-3.09065900	0.19882100	-1.66249400
C	-5.50890100	-0.06331200	0.68988900
H	-5.04971300	-0.26646300	2.78280800
H	-5.57558300	0.13675400	-1.45726000
H	-6.58160600	-0.09659200	0.83366200
N	0.61018200	-0.01306600	1.70627200
N	-0.60695400	0.00107300	1.71654600
N	3.46407400	-0.00033200	-0.67823500
N	-3.32094700	-0.12317300	1.67591300
C	0.71050400	1.24370200	-0.33707700
C	-0.68574800	1.26357000	-0.32364500
C	1.42897700	2.32589400	-0.82830800
H	2.51129500	2.31754200	-0.82767900
C	-1.37462500	2.37722300	-0.78885600
C	0.73565800	3.42624200	-1.32868400
H	-2.45560400	2.42241200	-0.74839400
H	1.28739900	4.27070200	-1.72499700
C	-0.65495300	3.45308600	-1.30580700
H	-1.18650300	4.32007000	-1.68046300

Figure S3e

TS8

C	-5.49853600	-0.55729000	-0.29981000
C	-5.02260800	-1.29202500	0.77668800
C	-3.64980000	-1.43096600	0.94054500
C	-2.80098200	-0.82525600	0.01350200
C	-4.57149600	0.00733200	-1.17176500
H	-6.55944300	-0.42175200	-0.46875900
H	-5.70617000	-1.75811900	1.47672900
H	-3.25203100	-2.01952400	1.75734500
H	-4.90484900	0.58972400	-2.02526300
C	-1.31894400	-0.95256100	0.13038200
C	-0.67240000	-1.19482000	1.36163900
C	0.69117900	-1.20729300	1.34677800
H	-1.23540300	-1.29087900	2.28123100
C	1.31721900	-0.98068900	0.10067400
H	1.27870100	-1.33629500	2.24720200
C	2.79998500	-0.85787300	-0.00957000
C	3.50519700	-0.15807000	0.97190800
C	4.88428900	-0.05242600	0.85415200
H	2.97811000	0.30990600	1.79529100
C	4.71801400	-1.31708500	-1.16281200
C	5.50857800	-0.64418500	-0.23631000
H	5.45831800	0.48854700	1.59749200
H	5.17135800	-1.79314900	-2.02674100
H	6.58101200	-0.58745900	-0.37434900
N	-0.65721500	-1.17931800	-1.04084500
N	0.63071400	-1.19332800	-1.05329500
N	-3.25440700	-0.11599200	-1.02521200
N	3.39408300	-1.42633900	-1.06106300
C	-0.62125500	1.35618600	0.01656100
C	0.64001700	1.35859100	0.00392600
C	-1.44769000	2.46188300	0.04433500
H	-2.53084700	2.45008200	0.04195500
C	1.45082100	2.47818600	0.01254400
C	-0.70570300	3.65624200	0.06429900
H	2.53455000	2.48233000	-0.01063500
H	-1.23763700	4.60160500	0.08585900
C	0.69651400	3.66443000	0.04738800

H	1.21771700	4.61595500	0.05522400

P8

C	1.49800200	-0.64181100	4.98373300
C	1.42139800	-0.75484700	3.60210800
C	0.53826600	0.07248700	2.90294200
C	-0.15740600	1.06913100	4.84739200
C	0.69388200	0.29147800	5.62522400
H	2.17154900	-1.27564500	5.54918900
H	2.02376200	-1.47793500	3.06568500
H	-0.80089800	1.81015400	5.31188300
H	0.71826500	0.41771900	6.70033400
C	0.47251000	0.00809200	1.41575100
C	1.64478000	0.09550300	0.70468500
C	1.64478000	0.09550300	-0.70468500
H	2.58523500	0.20707500	1.23313500
C	0.47251000	0.00809200	-1.41575100
H	2.58523500	0.20707500	-1.23313500
C	0.53826600	0.07248700	-2.90294200
C	1.42139800	-0.75484700	-3.60210800
C	1.49800200	-0.64181100	-4.98373300
H	2.02376200	-1.47793500	-3.06568500
C	-0.15740600	1.06913100	-4.84739200
C	0.69388200	0.29147800	-5.62522400
H	2.17154900	-1.27564500	-5.54918900
H	-0.80089800	1.81015400	-5.31188300
H	0.71826500	0.41771900	-6.70033400
N	-0.24239200	0.96852300	-3.52074600
N	-0.24239200	0.96852300	3.52074600
C	-0.76693100	-0.14287900	0.71376300
C	-0.76693100	-0.14287900	-0.71376300
C	-1.99732400	-0.35123800	1.39617300
H	-2.00511200	-0.36523400	2.47769100
C	-1.99732400	-0.35123800	-1.39617300
C	-3.16470000	-0.54405900	0.70614600
H	-2.00511200	-0.36523400	-2.47769100
H	-4.09092000	-0.70693600	1.24523700

C	-3.16470000	-0.54405900	-0.70614600
H	-4.09092000	-0.70693600	-1.24523700
